# Towards optimized methodological parameters for maximizing the behavioral effects of transcranial direct current stimulation

**DOI:** 10.3389/fnhum.2024.1305446

**Published:** 2024-07-02

**Authors:** Tyler Santander, Sara Leslie, Luna J. Li, Henri E. Skinner, Jessica M. Simonson, Patrick Sweeney, Kaitlyn P. Deen, Michael B. Miller, Tad T. Brunye

**Affiliations:** ^1^Institute for Collaborative Biotechnologies, University of California, Santa Barbara, Santa Barbara, CA, United States; ^2^Department of Psychological and Brain Sciences, University of California, Santa Barbara, Santa Barbara, CA, United States; ^3^U. S. Army DEVCOM Soldier Center, Natick, MA, United States; ^4^Center for Applied Brain and Cognitive Sciences, Tufts University, Medford, MA, United States

**Keywords:** non-invasive brain stimulation, transcranial direct current stimulation, meta-analysis, meta-regression, research methods

## Abstract

**Introduction:**

Transcranial direct current stimulation (tDCS) administers low-intensity direct current electrical stimulation to brain regions via electrodes arranged on the surface of the scalp. The core promise of tDCS is its ability to modulate brain activity and affect performance on diverse cognitive functions (affording *causal* inferences regarding regional brain activity and behavior), but the optimal methodological parameters for maximizing behavioral effects remain to be elucidated. Here we sought to examine the effects of 10 stimulation and experimental design factors across a series of five cognitive domains: motor performance, visual search, working memory, vigilance, and response inhibition. The objective was to identify a set of optimal parameter settings that consistently and reliably maximized the behavioral effects of tDCS within each cognitive domain.

**Methods:**

We surveyed tDCS effects on these various cognitive functions in healthy young adults, ultimately resulting in 721 effects across 106 published reports. Hierarchical Bayesian meta-regression models were fit to characterize how (and to what extent) these design parameters differentially predict the likelihood of positive/negative behavioral outcomes.

**Results:**

Consistent with many previous meta-analyses of tDCS effects, extensive variability was observed across tasks and measured outcomes. Consequently, most design parameters did not confer consistent advantages or disadvantages to behavioral effects—a domain-general model suggested an advantage to using within-subjects designs (versus between-subjects) and the tendency for cathodal stimulation (relative to anodal stimulation) to produce reduced behavioral effects, but these associations were scarcely-evident in domain-specific models.

**Discussion:**

These findings highlight the urgent need for tDCS studies to more systematically probe the effects of these parameters on behavior to fulfill the promise of identifying causal links between brain function and cognition.

## 1 Introduction

Non-invasive brain stimulation (NIBS) involves the introduction of exogenous energy (including magnetic, electrical, or ultrasonic) to cortical and subcortical brain regions to modify neuronal activity and draw causal links between regional brain function, cognition, emotion, and behavior (Dayan et al., 2013; Bestmann et al., 2015; Polania et al., 2018). Transcranial direct current stimulation (tDCS) is one of the most popular and widely-used NIBS methods, offering the ability to induce transient and subthreshold modulation of neuronal membrane potentials, with downstream consequences for the activity of neuronal populations and neural networks, neuroplasticity, and/or behavior (de Berker et al., 2013; Bestmann et al., 2015). While tDCS is a promising neuromodulatory tool, the precise mechanisms by which it exerts effects on physiology and behavior are still being discovered (Bikson et al., 2013; Dayan et al., 2013; Bestmann et al., 2015; Bikson et al., 2019). Emerging mechanistic models suggest that tDCS induces shifts of neuronal membrane potentials; that the nature of this shift (i.e., hyperpolarizing, depolarizing) is related to the orientation of neurons relative to the induced electrical field; that tDCS can induce large-scale oscillatory changes of brain activity; and that lasting after-effects of tDCS are related to changes in synaptic plasticity (Lafon et al., 2017; Bikson et al., 2019; Nitsche et al., 2019). However, much uncertainty still exists about how electrical current propagates through human tissue, which structures are effectively modulated by various electrode montages, how to effectively individualize stimulation protocols, and how various study design features might influence tDCS effects on observed outcomes (Datta et al., 2012; de Berker et al., 2013; Bestmann et al., 2015).

Ambiguity regarding tDCS mechanisms of action can make it difficult for researchers to select from myriad methodological parameters when designing experiments. An *a priori* review of related literature can complicate the issue due to heterogeneity of stimulation procedures and observed outcomes across studies, laboratories, and cognitive domains (Datta et al., 2012; Horvath et al., 2014; Bestmann et al., 2015). Indeed, published tDCS research varies widely in stimulation methods, study designs, and statistical methods employed; variation along each of these dimensions is likely to shape the effects of tDCS on brain physiology and behavior, and they likely interact in yet unknown ways. Herein we describe a meta-analytic, meta-regression modeling approach intended to provide an initial breadth-first understanding of relationships between methodological parameters employed in tDCS studies and behavioral outcomes across five cognitive domains, focusing only on studies examining healthy, neurotypical participants. We focused our analysis on 10 parameters of interest, including 7 stimulation-specific parameters and 3 experimental parameters (detailed in Table 1). These parameters were selected because they were relatively well represented in the literature across our outcome domains.

**Table 1:** The 10 independent variables considered under our meta-regression models, including the category, name, and a brief description of each.

Parameter category	Parameter name	Description
Stimulation	Target	The intended brain target of the stimulation electrode montage.
Stimulation	Laterality	The laterality of the brain target (i.e., left or right hemisphere, or bilateral stimulation of homotopic regions).
Stimulation	Electrode count	The number of stimulation electrodes used in the experiment, ranging from 2 to 10.
Stimulation	Individualization	Whether the experiment used a standardized or participant-specific electrode montage (i.e., MRI-guided targeting).
Stimulation	Intensity	The raw intensity of stimulation applied, ranging from 0.25 to 5 mA.
Stimulation	Polarity	Whether anodal or cathodal stimulation was applied.
Stimulation	Duration	The duration of stimulation applied, ranging from 5 to 30 min.
Experimental	Timing	Whether stimulation was administered prior to or during task execution (or some combination thereof).
Experimental	Session count	The number of stimulation sessions completed by participants, ranging from 1 to 20.
Experimental	Design	Whether the experiment used a within-subjects or between-subjects design.

The scope of our approach builds upon prior reviews and meta-analyses that are often focused on specific cognitive domains and behavioral outcomes, or alternatively, on specific neural/physiological responses to stimulation. Mixed or otherwise inconclusive patterns of results have been well-characterized across *many* disparate areas of neurostimulation research (Koenigs et al., 2009; Loo et al., 2010; Jacobson et al., 2012; Loo et al., 2012; Brunoni and Vanderhasselt, 2014; Lopez-Alonso et al., 2014; Wiethoff et al., 2014; Chew et al., 2015; Horvath et al., 2015a,b; Lopez-Alonso et al., 2015; Dedoncker et al., 2016; Hill et al., 2016; Horvath et al., 2016a,b; Mancuso et al., 2016; Vannorsdall et al., 2016; Heroux et al., 2017; Imburgio and Orr, 2018; Loo et al., 2018; Oldrati and Schutter, 2018; Rassovsky et al., 2018; Falcone et al., 2019; Schroeder et al., 2020; Wischnewski et al., 2021). However, the most popular approach to quantifying and describing heterogeneity is—understandably—rather piecemeal. Past studies addressing potential moderating factors (e.g., the polarity of stimulation, or the laterality of a target region) typically either parse out effects for separate analyses (e.g., looking at trends for left vs. right hemisphere stimulation independently, rather than modeling the contrast) or perform meta-regression based on small subsets of features (Jacobson et al., 2012; Brunoni and Vanderhasselt, 2014; Dedoncker et al., 2016; Hill et al., 2016; Mancuso et al., 2016; Imburgio and Orr, 2018; Oldrati and Schutter, 2018; Schroeder et al., 2020). Our goal, in contrast, is to consider a wider breadth of tasks, experiment parameters, and domains to explicitly quantify systematic differences in behavioral outcomes associated with these design factors.

### 1.1 Stimulation parameters

One critical consideration for experimenters is determining the arrangement of electrodes on the scalp to effectively target a brain region of interest. When doing so, experiments tend to rely upon methods demonstrated as effective in extant research, and/or on results derived from models of predicted current flow (Datta et al., 2009; Bikson et al., 2012, 2013; Ramaraju et al., 2018; Rich and Gillick, 2019). Researchers typically arrange electrodes on the scalp in reference to an anatomical coordinate system, such as the International 10–20 System (DaSilva et al., 2011), and/or based on outcomes from neuronavigation or peak activations identified in functional tests (e.g., motor-evoked potentials). In all cases, the intent is to maximize current density at a brain target believed to be functionally-relevant for a cognitive process or behavioral outcome of interest (Miranda et al., 2009; Sadleir et al., 2012; Rich et al., 2017). The target brain regions identified in our review included the dorsolateral prefrontal cortex (DLPFC), posterior parietal cortex (PPC), and primary motor cortex (M1), as well as a number of other diverse stimulation targets spanning nearly the whole brain.

The targeted brain regions were variably lateralized, with most reported effects (418 out of 721 total) localized to the left hemisphere and a minority of effects derived through bilateral stimulation of homotopic regions (59 total). When referring to laterality of stimulation, it is typically the case that the anodal electrode is placed over the targeted region (e.g., left DLPFC), and a reference cathodal electrode is placed over the contralateral hemisphere (e.g., right supraorbital area) or on an ipsilateral or contralateral extracephalic area (e.g., shoulder, bicep). While often treated as a relatively trivial decision in practice, it is important to realize that the placement of the cathode likely carries important implications for physiological and behavioral influences of tDCS (Bikson et al., 2010).

Most studies we identified used two saline-soaked sponges, one anode and one cathode, to administer tDCS; other studies used multi-electrode montages that are assumed to provide higher focality and maximize current density at both superficial and relatively medial targets (Datta et al., 2009). We also found that most studies tended to use a consistent electrode positioning across individuals, whereas a minority used individualized montages based on structural and/or functional magnetic resonance imaging (MRI) scans. Individualized montages are becoming more commonplace and are thought to compensate for varied brain morphology, functional neuroanatomy, and head sizes and shapes (Datta et al., 2012; Kim et al., 2014; Woods et al., 2016).

Stimulation procedures often involve a slow ramp-up (e.g., over a 30 s window) to a target intensity, maintaining that peak intensity level for a specific duration (e.g., 20 min), and then a slow ramp-down to terminate stimulation (Nitsche and Paulus, 2000; Gandiga et al., 2006). Peak stimulation intensity is typically between 0.5–2.0 milliamperes (mA; with the conventional limit being 2 mA) (Nitsche and Bikson, 2017), with one meta-analysis demonstrating a mean tDCS stimulation intensity of 1.5 mA when considering studies done in the cognitive domain (Jacobson et al., 2012). Modeling and experimental work suggests that stimulation intensity can have paradoxical, nonlinear effects on neuronal activity and behavioral effects of tDCS (Batsikadze et al., 2013; Bonaiuto and Bestmann, 2015; Esmaeilpour et al., 2018). For example, 1 mA stimulation intensity can produce hyperpolarization whereas 2 mA can produce depolarization of motor neurons (Batsikadze et al., 2013). One meta-analysis suggests that on the aggregate, higher intensity stimulation (operationalized as current density under the anode) is more likely to benefit task accuracy during DLPFC stimulation (Dedoncker et al., 2016). In our review, however, we focused primarily on raw stimulation intensity (as opposed to current density) and found studies administering stimulation as low as 0.25 mA intensity and as high as 5 mA.

The duration of peak stimulation intensity is typically between 5 and 30 min (as seen in our review), with one meta-analysis demonstrating an average tDCS stimulation duration of 15.2 min when considering studies performed in cognitive domains (Jacobson et al., 2012). Some of the nonlinear responses found with stimulation intensity can interact in unexpected ways with stimulation duration. For example, 1 mA of tDCS can induce depolarization during the first 13 min of stimulation, but hyperpolarization during a latter 13 min of stimulation (Monte-Silva et al., 2013).

A further critical decision point in designing a tDCS study is whether to deliver anodal or cathodal stimulation to the target site. Some mechanistic models of tDCS, namely *sliding scale* models, suggest that stimulation polarity influences whether tDCS can increase or decrease neural activity (Bikson et al., 2013; Bestmann et al., 2015). Specifically, neuronal populations residing in brain regions underlying the anode will experience depolarization, and populations under the cathode will experience hyperpolarization. This simplistic model has been repeatedly challenged (de Berker et al., 2013; Bestmann et al., 2015; Jackson et al., 2016; Bestmann and Walsh, 2017), but remains as a strong motivator for scientists who compare (experimentally and statistically) anodal versus cathodal stimulation over a target brain region, typically in reference to a sham condition.

Thus, stimulation parameters are variably selected and applied across studies. It is known that variation of certain parameters can elicit unexpected and nonlinear effects on brain physiology and behavior, and it is likely that many of these parameters interact in ways that have yet to be discovered. Here we focus primarily on additive, main effects due to the combinatorial explosion in model parameter space when exploring interactions between our 10 predictors of interest. However, it is still of central interest to uncover how these various factors might jointly-modulate one another in any given neurostimulation experiment.

### 1.2 Experimental and statistical parameters

In addition to stimulation parameters, our model also considered the influence of 3 experimental design parameters. Across studies, tDCS is variably administered either before task performance (offline), during task performance (online), or a mixture of both (offline-online) (Woods et al., 2016; Imburgio and Orr, 2018). Original research and meta-analyses suggest that offline stimulation is more likely than online stimulation to enhance performance on working memory tasks (Hill et al., 2016; Friehs and Frings, 2019; Zivanovic et al., 2021), but that the opposite might be true for skill acquisition (Martin et al., 2014) and visuospatial tasks (Oldrati et al., 2018). In our review, we indeed found that offline stimulation was employed more frequently than online stimulation (347 vs. 276 effects, respectively), with comparatively fewer studies using a mixed-timing design. Furthermore, while many studies use a single-session design involving only one (e.g., 20 min) administration of tDCS during a single visit to the laboratory, some studies involve the repeated administration of tDCS over the course of successive visits. While repeated sessions of tDCS are a common design feature in clinical research and are associated with higher treatment efficacy relative to single-session studies (Fregni et al., 2006; Boggio et al., 2007; Loo et al., 2010, 2012), they are used less frequently in studies with healthy neurotypical adults. When such a design is used, it is typically in the context of studies examining the effects of tDCS on cognitive training or skill acquisition paradigms. In general, it is thought that repeated tDCS can facilitate memory consolidation (Reis et al., 2009; Marshall et al., 2011), and meta-analyses suggest there may be insufficient evidence to evaluate any potential advantage of repeated- versus single-session tDCS on cognitive function (Horvath et al., 2015a).

In tDCS studies, within-participants designs are generally preferred to between-participants design given the inherent challenges associated with inter-participant variation in tDCS experiments, including factors such as the brain’s morphology and function, genetic attributes, consumption patterns (e.g., caffeine), menstrual cycle, aptitudes, and other trait-based variables (Lopez-Alonso et al., 2015; Vergallito et al., 2022). They are also considered the more powerful design for detecting differences among conditions because analyses partition out between-participants variance and reduce error (Kirk, 2013). That said, many studies use between-participants designs, typically for scheduling efficiency (tDCS sessions are typically separated by at least 24 h), to avoid carryover effects, or because the associated large sample size helps afford analyses of individual differences. Some studies balance power, efficiency, and scale by using mixed designs, which include one or more within- *and* between-participants factors. However, estimation of mixed effects, particularly in the context of the models described here (which generally focus on binary comparisons between conditions rather than factorial comparisons), is less straightforward. We therefore focus on studies using *either* a between-subjects or within-subjects design, which were represented fairly equivalently across the domains considered in our review.

### 1.3 Performance domains

The meta-analytic model was designed to understand how the 10 stimulation and experimental parameters are associated with performance outcomes across 5 cognitive domains, including vigilance, working memory, visual search, response inhibition, and motor performance. We chose these five domains because they have all received considerable attention in the scientific literature and exhibit variability in the brain regions targeted, the stimulation methods, and/or the experimental and statistical methods employed. Moreover, and perhaps most importantly, these 5 domains tend to underlie performance in many applied settings of interest including driving, aviation, and military.

Vigilance refers to a sustained focus of attention on stimulus detection tasks over lengthy periods of time (Parasuraman, 1976; Warm et al., 2008; Hancock, 2017), such as when continuously monitoring a radar screen. Maintaining vigilance is stressful and places high demands on attentional resources, resulting in vigilance decrements (i.e., reduced detection of critical stimuli) over time (Grier et al., 2003; Warm et al., 2008). Given its reliance on arousal and alertness, sustained attention, and information processing, many brain systems have been implicated in vigilance performance. These include the prefrontal cortex, reticular formation, thalamus, and basal forebrain cholinergic system (Parasuraman et al., 1998). In general, anodal tDCS targeting the DLPFC appears to reduce vigilance decrements (McIntire et al., 2014; Nelson et al., 2014), though one study showed that the same tDCS increased the rate of mind wandering during a vigilance task (Axelrod et al., 2015). From our review of the literature, we included 93 effects derived from tDCS experiments on vigilance, primarily involving DLPFC stimulation.

Working memory refers to mechanisms responsible for temporarily storing, processing, and manipulating task-relevant information in memory (Baddeley, 1992; Miyake and Shah, 1999). Working memory plays a pervasive role in daily life and is a critical process underlying performance on planning, reasoning and problem solving, and decision-making tasks (Kyllonen and Christal, 1990; Davidson and Sternberg, 2003; Hinson et al., 2003; Gilhooly, 2004). It has also been a topic of interest among cognitive neuroscientists interested in mapping working memory processes to brain regions and networks, which has found strong evidence that the lateral prefrontal cortex is involved in the temporary maintenance of task-relevant information, and that the distribution of brain activity across widespread networks is dependent on many task-related parameters such as the sensory modality being used (e.g., visual, auditory), the nature of stimuli (e.g., verbal, spatial, motor, faces) being maintained or manipulated, and whether the information is retrospective or prospective (D'Esposito, 2007). In general, the prefrontal cortex appears to be a critical node in a distributed working memory network that coordinates the involvement of other brain regions more specialized in specific functions (e.g., sensory, representational, and action-related) (Postle, 2006). The effects of tDCS on working memory performance have engendered some debate in the scientific literature, with some meta-analyses suggesting improvement of working memory (in accuracy or response times) with anodal tDCS targeting the left or right DLPFC (Brunoni and Vanderhasselt, 2014; Dedoncker et al., 2016; Hill et al., 2016; Mancuso et al., 2016), and another meta-analysis suggesting no evidence for improvement (Horvath et al., 2015a). From our review of the literature, we included 115 effects of tDCS on working memory, also primarily involving PFC stimulation.

Visual search refers to the process of finding a visual target among distractors, and is typically assumed to involve interactions between preattentive processing and focal attention (Wolfe, 2010; Eckstein, 2011; Chan and Hayward, 2013). Visual search is extremely common in applied and daily tasks, such as searching for a weapon in luggage, finding lung nodules on a radiograph, identifying suspects in a crowd, or simply finding a matching pair of socks (Eckstein, 2011). It also recruits a wide range of brain regions including ventral and dorsal regions of the prefrontal cortex (and frontal eye fields; FEF) (Anderson et al., 2007), multiple areas of the parietal cortex (Donner et al., 2000), and the occipital cortex (Nobre et al., 2003). Studies suggest that anodal and cathodal tDCS over the left FEF can improve target detection during a visual search task (Nelson et al., 2015), that cathodal stimulation of the right posterior parietal cortex (but not FEF) can reduce the benefits of practice in a visual search task (Ball et al., 2013), and that anodal stimulation of the right inferior frontal or posterior parietal cortex can enhance performance on a task involving searching for threats in complex scenes (Falcone et al., 2012; Callan et al., 2016). From our review of the literature, we included 176 tDCS effects on visual search, primarily involving PFC and parietal stimulation.

Inhibitory control refers to the ability to suppress thoughts or behaviors that are not relevant or conducive to task performance, and is considered a critical executive function that enables adaptive behavior (Chambers et al., 2009; Congdon et al., 2012). Response inhibition enables the adaptive control of cognitive processes and motor behavior, and failure of response inhibition is through to underlie both clinical and subclinical impulsivity (Horn et al., 2003). In applied and daily tasks, response inhibition enables people to inhibit prepotent behavioral responses across a range of settings—such as firing or not firing a weapon (Biggs et al., 2015), swinging or not swinging a baseball bat (Nakamoto and Mori, 2012), or not eating a second piece of cake (Appelhans, 2009). Dominant theories of inhibitory control emphasize the role of several regions of the prefrontal cortex, including the dorsolateral, inferior, and orbital frontal cortex (Duncan and Owen, 2000; Aron et al., 2004). While some debate exists regarding the modular versus distributed roles of prefrontal brain regions in inhibitory processes (Hampshire and Sharp, 2015), particular emphasis has been given to the right inferior frontal gyrus given results of functional neuroimaging, lesion, and transcranial magnetic stimulation (TMS) studies (Aron et al., 2004, 2014). With tDCS, a recent meta-analysis demonstrated generally small but significant effect of tDCS on inhibitory control outcomes (including go−/no-go task and stop-signal task), especially with right inferior frontal gyrus stimulation (Schroeder et al., 2020). From our review of the literature, we included 84 effects on inhibitory control, primarily involving PFC stimulation.

Finally, motor performance here refers to the process of acquiring new abilities to perform novel sequences of skilled behaviors to accomplish a goal, from typing on a keyboard to riding a bike. Acquiring a new skill relies upon experience-dependent neuroplasticity in the brain, often tied to practice and consolidation (Karni et al., 1998; Robertson et al., 2004), which can occur over the course of hours, days, or weeks (Korman et al., 2003). Neuroplastic changes associated with motor skill acquisition are often considered the locus of the primary motor cortex (Karni et al., 1995, 1998; Sanes and Donoghue, 2000). In addition to the motor cortex, the cerebellum has received attention due to its potential involvement in the initiation of limb movements and the improvement of motor skills (Gilbert and Thach, 1977; Houk et al., 1996; Thach, 1996; Kitazawa et al., 1998). Studies using tDCS to influence motor skill acquisition variably target the primary motor cortex and cerebellum. Reviews and meta-analyses suggest that anodal tDCS targeting the primary motor cortex can improve motor learning and motor function (Reis and Fritsch, 2011), and both anodal and cathodal tDCS targeting the cerebellum can accelerate motor learning, motor adaptation, and procedural learning (Oldrati and Schutter, 2018). This domain represented the largest in our survey of the literature, spanning 253 effects related to motor and procedural skill acquisition.

## 2 Methods

### 2.1 Study search and selection

A literature search was performed by authors S.L. and P.S. using PsycINFO, PubMed, Google Scholar, and the tDCS Database.^1^ The key search terms were [tDCS] or [HD-tDCS] in combination with any of the following domain-specific terms: [motor skill], [motor learning], [procedural learning], [implicit learning], [skill learning], [working memory], [vigilance], [endurance], [visual search], and [inhibition]. Unions of key terms (using the AND function) were queried using the options for All Fields or Any Field for three of the selected databases (PubMed, PsycINFO, and the tDCS Database); when searching Google Scholar, key search terms were queried in the full text, and due to the high search yield only the first 300 results were included for screening. Searches were not restricted by publication date.

**Figure 1:**
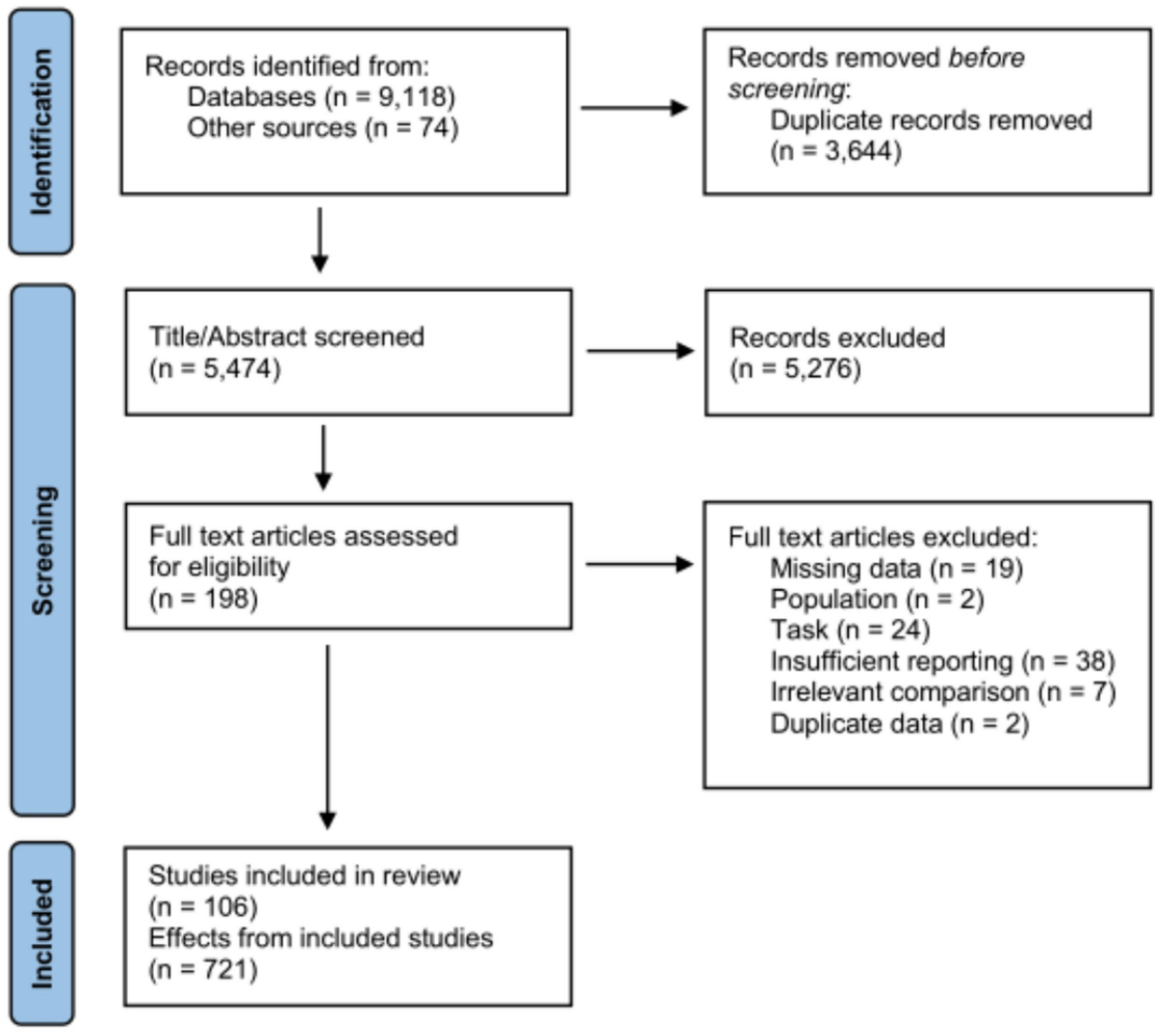
Summary flowchart of literature search and screening procedure.

Because we sought to model specific effects of experimental design parameters on behavioral performance outcomes, we applied strict inclusion criteria to narrow down relevant studies. Beyond the basic requirement of publication in a peer-reviewed scientific journal, these included: (1) no studies focused exclusively on minors, older adults, or individuals with neuropsychiatric conditions (we extracted *only* effects related to healthy younger adults in studies based on these comparisons); (2) application of tDCS and not tACS/tRNS (although again, in cases where these were compared, we extracted *only* tDCS-related effects); (3) ability to extract simple within-subject or between-subject effects; (4) binary comparisons between either anodal or cathodal stimulation vs. sham stimulation (i.e., no anodal vs. cathodal comparisons, or comparisons between active stimulation conditions across different intensities); (5) stimulation targeted over a specific brain region (e.g., focused on DLPFC alone rather than a ‘network-like’ approach to neuromodulation, exciting both frontal and parietal areas); (6) reporting of behavioral outcomes (i.e., we did not include effects on fMRI or EEG-derived brain activity); and (7) sufficiently clear reporting of all stimulation parameters/procedures. This yielded 42 studies in the motor domain (253 total effects) (Antal et al., 2004; Boggio et al., 2006; Vines et al., 2008; Tecchio et al., 2010; Leite et al., 2011; Ferrucci et al., 2013; Saucedo Marquez et al., 2013; Vollmann et al., 2013; Furuya et al., 2014; Prichard et al., 2014; Zimerman et al., 2014; Cantarero et al., 2015; Minarik et al., 2015; von Rein et al., 2015; Ambrus et al., 2016; Doppelmayr et al., 2016; Ehsani et al., 2016; Naros et al., 2016; Fan et al., 2017; Filmer et al., 2017a; Focke et al., 2017; Fujiyama et al., 2017; Hashemirad et al., 2017; Pixa et al., 2017; Rumpf et al., 2017; Samaei et al., 2017; Apsvalka et al., 2018; Dumel et al., 2018a,b; Ballard et al., 2019; Fehring et al., 2019; Jin et al., 2019; Shilo and Lavidor, 2019; Spampinato et al., 2019; Talimkhani et al., 2019; Pollok et al., 2020; Rocha et al., 2020; Ballard et al., 2021; Nguemeni et al., 2021; Parma et al., 2021; Sevilla-Sanchez et al., 2021; Toth et al., 2021), 18 visual search studies (176 effects) (Bolognini et al., 2010; Clark et al., 2012; Coffman et al., 2012; Ball et al., 2013; Ellison et al., 2014; Cosman et al., 2015; Nelson et al., 2015; Reinhart and Woodman, 2015; Callan et al., 2016; Ellison et al., 2017; Filmer et al., 2017a,b; Falcone et al., 2018; Lanina et al., 2018; Nydam et al., 2018; Sung and Gordon, 2018; Grasso et al., 2020; Wagner et al., 2020), 16 working memory studies (115 effects) (Boehringer et al., 2013; Zwissler et al., 2014; Nikolin et al., 2015; Au et al., 2016; Chrysikou et al., 2017; Naka et al., 2018; Abellaneda-Perez et al., 2019; Ankri et al., 2020; Baumert et al., 2020; Hussey et al., 2020; Murphy et al., 2020; Assecondi et al., 2021; Karthikeyan et al., 2021; Pupikova et al., 2021; Zivanovic et al., 2021; Caulfield et al., 2022), 15 vigilance studies (93 effects) (Plewnia et al., 2013; Manuel et al., 2014; McIntire et al., 2014; Nikolin et al., 2015; Hanken et al., 2016; Ironside et al., 2016; McIntire et al., 2017; Borragan et al., 2018; Brunnauer et al., 2018; Jacoby and Lavidor, 2018; Naka et al., 2018; Filmer et al., 2019; Coulborn et al., 2020; Luna et al., 2020; van Schouwenburg et al., 2021), and 15 inhibition studies (84 effects) (Hammer et al., 2011; Gladwin et al., 2012; Plewnia et al., 2013; Oldrati et al., 2016; Gomez-Ariza et al., 2017; Friedrich and Beste, 2018; Angius et al., 2019; Boudewyn et al., 2019; Denis et al., 2019; Dubreuil-Vall et al., 2019; Edgcumbe et al., 2019; Kuehne et al., 2019; Baumert et al., 2020; Hussey et al., 2020; Thomas et al., 2020). A summary flowchart of search and screening procedures is shown in Figure 1; the studies included in each domain are listed for convenience in Supplementary Table S1. We note that this represents only a subset of all published studies under each domain (some of which employed multiple tasks capturing various cognitive constructs) and is not intended to be exhaustive; however, we suggest the total number of effects considered affords a reasonable estimation of how various stimulation and study design parameters might modulate behavior.

### 2.2 Data extraction, effect size estimation, and parameter coding

Whenever possible, we extracted exact descriptive statistics (means and standard deviations, or standard errors) from the reported text. However, in many cases, this information was only presented graphically. We therefore used the WebPlotDigitizer^2^ to convert graphical displays into numerical data. Effects were excluded here if error bars were visually indistinguishable and/or if their endpoints could not be reliably identified. For each study, we attempted to extract as much behaviorally-relevant information as possible, particularly when multiple effects were reported across different experiment blocks or outcome variables (e.g., accuracy and/or reaction time), allowing us to better capture variability in the effects of tDCS on performance—which may be transient over the course of a task. Irrelevant, non-task-related measures were excluded (e.g., self-reported side effects), as were ancillary task-related measures that did not inform the cognitive domains of interest (e.g., for inhibition, we excluded reaction times to ‘congruent’ trials in Stroop tasks).

Effect sizes and their standard errors were estimated using Hedges’ *g*. For between-subject designs where group means, standard deviations, and sample sizes are known, this is trivial; however, for within-subject designs, the standard deviation around the mean difference depends upon the correlation between repeated measures. No study in our survey of the literature provided this information. We ultimately chose to assume a moderate correlation (*r* = 0.50) for all within-subject effects (equivalently, taking an average of the variances)—a common practice in power analyses when the within-subject correlation is not known *a priori*. Furthermore, to enable more generalized assessments of behavioral outcomes regardless of the exact task paradigm or behavioral construct being measured, each effect size was assigned a positive or negative sign depending on whether the relevant comparison indicated an improvement or detriment to behavior, respectively. For example, faster reaction times during active stimulation vs. sham were assigned a positive sign, whereas higher error rates earned a negative sign. Thus, under the model, we could assess whether certain experiment design parameters conferred positive or negative effects *on average* for a given domain, irrespective of specific tasks or outcomes.

Each effect was coded alongside the 10 stimulation and experiment parameters summarized in Table 1. The stimulation target parameter was given as the intended brain region targeted by the electrode montage. In cases where some brain regions were poorly-represented (i.e., were only targeted in one or two studies, or constituted an exceptionally small proportion of the total number of effects), we attempted to collapse them into an ‘Other’ category grouped with other anatomically-proximal regions. Target laterality was coded as either Left, Right, or Bilateral (in the latter case, when stimulation was applied to homotopic regions in both the left and right hemisphere). We also recorded the number of electrodes used in the montage and whether the placement of electrodes was individualized (e.g., guided by an anatomical MRI for each subject; coded as a binary Yes or No). Individualized target montages were exceedingly rare, occurring primarily in the motor performance domain. Raw stimulation intensity was recorded in milliamperes and polarity coded as either Anodal or Cathodal (always in comparison to a sham stimulation condition). The duration of stimulation was given in minutes and coded as Online (applied during task performance), Offline (prior to task performance), or Mixed (for cases when online stimulation was applied for only a portion of task performance). The total number of stimulation sessions completed by participants was also given for each effect. Finally, we recorded whether each comparison was made under a within-subjects or between-subjects framework. A summary of how these parameters were represented under each domain can be found in Supplementary Table S2.

As an additional supplementary analysis, we also attempted to extract or estimate current *density* over the target brain region (in mA / cm^2^). This was possible for all studies in included in the visual search and vigilance domains, but it resulted in some loss of data for the motor, working memory, and inhibition domains (due to, e.g., the use of customized or otherwise more complex multielectrode arrays for which it was not possible to reliably estimate current density). We found that this parameter did not inform variability in behavioral outcomes and generally exhibited much greater posterior uncertainty; thus, given our already-strict inclusion criteria, we do not use it in the models and results described below. For completeness, however, we visualize these models in Supplementary Figure S1.

### 2.3 Hierarchical Bayesian meta-regression: model specification and estimation

Given that nearly all studies reported multiple behavioral outcomes as a function of their tDCS manipulations, we used a hierarchical Bayesian meta-regression framework to model the average magnitude of behavioral effects associated with our design parameters of interest, accounting for both *intra-* and *inter*-study variability in the effects of neurostimulation across all reported comparisons. We elected to use a Bayesian framework over a comparable frequentist framework for several reasons. First, Bayesian methods afford probabilistic estimates in favor of hypotheses and the existence of an effect by computing full posterior distributions given the observed data, which in turn allow for more direct estimates of *uncertainty* surrounding an effect. Second, Bayesian methods allow us to be pragmatic in incorporating our prior beliefs (or perhaps alternatively, our lack of strong prior convictions) into a model and help us control for potential bias by retaining identical prior assumptions regardless of the data or cognitive domain. Finally, Bayesian methods allow for principled, probabilistic model comparison, useful for exploratory analyses and quantifying uncertainty in performance between models using different sets of parameters.

All models were specified and estimated using Stan^3^ and brms (Bürkner, 2017) in R. For consistency, and to avoid the possibility that any given model could be unduly influenced by arbitrary prior specifications, key parameters for all models were estimated under the same set of weakly-informative priors:


$$\mathrm{Intercept}\sim \mathrm{Normal}\;\left(0,1\right)$$

$$\mathrm{Slopes}\sim \mathrm{Normal}\;\left(0,2.5\right)$$

$$\mathrm{Random}\;\mathrm{effect}\;SD\sim \mathrm{Half}-\mathrm{Cauchy}\;\left(0,0.5\right)$$

Note that, because we obtained standard errors around each recorded effect size, we could incorporate these values directly into the model likelihoods, precluding the need to estimate noise variance as an additional free parameter. These priors were selected based on previous recommendations for Bayesian meta-analysis (Williams et al., 2018; Reis et al., 2023) with one notable change. Typically, a traditional meta-analysis only models an intercept (capturing the average effect size pooled across studies): a Normal(0, 1) prior for this term is arguably quite sensible in these situations (this assumes with 95% probability that the ‘true’ pooled effect lies somewhere between *g* = [−2, 2]). However, here we are not just pooling effect sizes on average, but also determining the extent to which effect sizes are systematically moderated by differences in experiment design factors (that is, we also need to model slopes). Given that we do not necessarily have a good *a priori* guess as to how, for example, effect size might differ on average between Anodal and Cathodal stimulation, we use a wider, weaker prior.

In constructing the design matrix for each model, categorical regressors (e.g., stimulation polarity) were coded using sum-to-zero deviation contrasts, which enable more traditional ‘ANOVA-like’ comparisons of mean differences between a given factor level and a reference level. Continuous regressors (e.g., stimulation intensity) were centered about their respective means. We further specified a random intercept term to account for variation in reported effects across studies. Random slopes were omitted, both to minimize model complexity (and thereby ensure convergence) and because many comparisons are empirically unidentifiable within a given study (e.g., many studies reported *either* anodal vs. sham or cathodal vs. sham stimulation designs, so one cannot estimate differential effects between the two on an individual study level). Thus, using Wilkinson notation, an additive model containing all predictors may be written as:


$$\begin{array}{l} \mathrm{hedgesG}|se\left(\mathrm{stdErrorG}\right)\sim \mathrm{stimPolarity}+\mathrm{targetRegion}\\ +\mathrm{individualizedTarget}+\mathrm{targetLaterality}+\mathrm{numSessStim}\\ +\mathrm{stimDurationPerSess}+\mathrm{stimIntensity}+\mathrm{stimOnlineOffline}\\ +\mathrm{numElectrodes}+\mathrm{designType}+\left(1|\mathrm{studyID}\right) \end{array}$$

Continuous predictors such as stimulation intensity imply a single slope, while categorical factors such as stimulation polarity imply *k* – 1 contrasts, where *k* is the number of factor levels (so, for example, the polarity term tests for differences associated with Anodal vs. Cathodal stimulation, whereas the stimulation timing term may imply two fit parameters: one for Offline vs. Mixed comparisons, and one for Online vs. Mixed).

Robust exploration of the posterior space for all models was performed via Hamiltonian Monte Carlo, with four independent chains of 15,000 iterations each (5,000 of which were used as warm-up samples). We ensured that all models properly converged to equilibrium for all parameters using classical benchmarks, including: the effective sample size (considering the autocorrelation between independent posterior draws); the Monte Carlo standard error (relative to the posterior SD); *R*-hat (the variance ratio between each chain relative to *all* chains); and no divergent Monte Carlo transitions after warm-up.

For statistical inference, we consider 95% credibility intervals (using the highest posterior density interval) around the posterior median parameter estimates along with the ‘probability of direction’ (*p*_d_), capturing the proportion of the posterior density above or below zero. Here, *p*_d_ can range from 0.50 (indicating that the posterior is centered at zero, i.e., half its density lies above zero and half below zero) to 1 (in the most extreme case, that *no* proportion of the posterior density intersects zero)—thus, values of *p*_d_ closer to 1 indicate more evidence for the presence of a directional/nonzero effect, and *p*_d_ > 0.95 can *roughly* be thought of as a Bayesian analogue to the frequentist *p*-value, such that less than 5% of the full posterior distribution intersects zero (Makowski et al., 2019). However, an important point here is that we do not wish to frame these results in terms of ‘statistical significance’, but rather a simple consideration of *evidence* for nonzero differences given the posterior densities for each parameter. Bayes factors are not reported due to their sensitivity to priors and because they often (regrettably) encourage the sorts of binary thinking about hypotheses that Bayesian methods are meant to circumvent. Instead, we compute a Bayesian analogue of Cohen’s *d* for categorical predictors by taking the posterior draws for each regressor and dividing them by the square root of the summed residual variances and random effects variances (as per convention for hierarchical linear models) (Westfall et al., 2014), yielding a posterior distribution for each non-continuous model-level effect. Finally, fit indices were computed using the conditional *R*^2^ over the full model (taking both fixed and random effects together), and the marginal *R*^2^ capturing variance that can be attributed to the fixed effects alone (Gelman et al., 2019).

### 2.4 Exploratory modeling of interaction effects

The models described above focused simply on additive, main effects for each predictor of interest—we did not have any *a priori* hypotheses related to how these various experiment design parameters might interact to produce differential behavioral outcomes. However, it is likely that interaction effects *do* exist between these factors. One general challenge with modeling these potential effects in an exploratory fashion is combinatorial explosion: given the number of parameters under consideration, the possible model space is massive and may include models with many higher-order interactions that are uninterpretable.

In an effort to perform an exploratory search in a restricted, albeit principled and maximally-unbiased fashion, we focused on models containing either two-way or three-way interactions (the latter of which included all three marginal two-way interactions) constructed on a dataset comprised of *all* domains combined. This would allow us to identify interaction models that appear to generalize across domains and subsequently test the extent to which they inform domain-specific models. To assess the performance of candidate models against the ‘base model’ (i.e., solely including additive, main effects), we used Bayesian approximate leave-one-out cross validation with Pareto-smoothed importance sampling (PSIS) (Vehtari et al., 2017; Paananen et al., 2021; Vehtari et al., 2024). In brief, this approach takes advantage of our fully Bayesian modeling framework to circumvent typically-costly cross-validation routines requiring many model re-fits, instead using PSIS to imagine the likelihood our model would assign to each datapoint *as if* they were held-out, yielding a new cross-validated posterior predictive distribution over the data (a *pointwise predictive density*). We can then directly quantify the difference in predictive performance between candidate and base models by comparing their mean, expected log pointwise predictive densities (*ELPD*s), which has an associated standard error: the ratio of the *ELPD* differential to the standard error of the difference (akin to a *Z*-statistic) is a simple summary metric that can inform us whether the addition of putative interaction terms significantly improves predictive performance above the base, additive model (a fairly liberal criterion is to check whether the absolute value |*ELPD_Diff_* / *SE_Diff_*| > 2, similar to a two-tailed test at α = 0.05).

Under this framework, we first performed a comprehensive sweep over all possible inclusions of a single two-way interaction term (28 models total). None of these candidate models, however, suggested a significant improvement in predictive performance above the base model (Supplementary Table S3). We then performed a similarly exhaustive sweep over 56 candidate three-way interaction models, and here, there were three models that suggested significant improvement over the base model (Supplementary Table S4), each with the following terms: (1) Design Type (Within- vs. Between-Subjects) × Number of Electrodes in Montage × Stim Intensity (mA); (2) Design Type × Number of Electrodes × Stim Duration per Session (min); and (3) Design Type × Number of Electrodes × Stim Duration. Notably, there was considerable overlap in the relevant factors for each candidate interaction—we therefore fit a four-way interaction model that provided, at best, a marginal improvement over the base model and did not significantly outperform any of the individual three-way models (Table 2).

**Table 2:** Differences in candidate model predictive performance derived via Bayesian approximate leave-one-out cross-validation.

Candidate model	*ELPD_Diff_*	*SE_Diff_*	*ELPD_Diff_ / SE_Diff_*
Design Type × Stim Intensity × Stim Duration × Num Electrodes	—	—	—
Design Type × Num Electrodes × Stim Intensity	−10.984	7.330	−1.499
Design Type × Stim Intensity × Stim Duration	−16.034	10.357	−1.548
Design Type × Num Electrodes × Stim Duration	−18.334	10.245	−1.790
Base Model (Only Additive Terms)	−33.532	16.581	−2.022

The four-way interaction model provided the best performance overall and thus differences in fit are presented with respect to this model. *ELPD_Diff_* gives the mean difference in the expected log pointwise predictive densities (negative values shown here indicate decrements in predictive performance relative to the top model); *SE_Diff_* gives the standard error of the difference. The top model significantly differs in performance from any other model where |*ELPD_Diff_* / *SE_Diff_*| > 2.

Because the four-way model did not confer a particular advantage over any individual three-way model, we excluded it from further testing and instead consider the various three-way models noted above to be *competitive*. These were subsequently applied to each individual domain to assess the extent to which they may inform domain-specific differences in behavioral effects above and beyond their respective base, additive models.

## 3 Results

### 3.1 Motor performance

**Figure 2:**
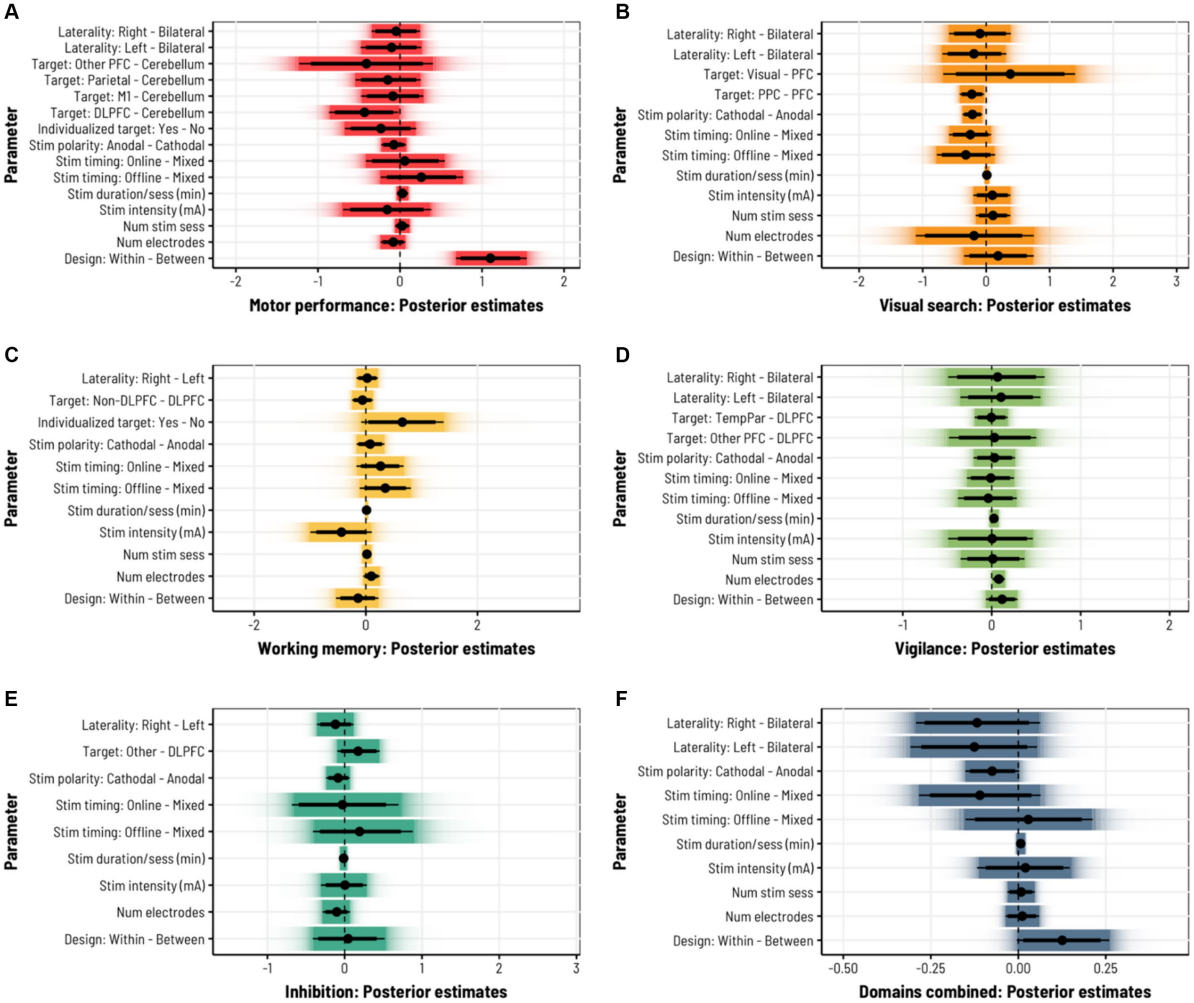
Contrasting estimates of multiple neurostimulation and experimental parameters on behavioral performance outcomes, both within-domain and between-domain. For categorical predictors (e.g., target laterality), contrasts were specified in an *A – B* fashion, such that level *B* served as the reference point. Thus, positive values suggest larger effect sizes (on average) for level *A* compared to *B*; negative values indicate the reverse. For continuous predictors (e.g., stimulation intensity), positive values suggest larger effect sizes with increasing values of the predictor (e.g., stronger stimulation dosages); negative values suggest the inverse association. Values closer to zero indicate weak or no association with performance outcomes, either at the level of mean differences between factor levels or a linear relationship with continuous predictors. Point intervals give 90–95% credibility intervals (i.e., highest density intervals) around the median posterior estimates (thick lines = 90% CI; thin lines = 95% CI; solid gradient areas indicate the 95% CI). **(A)** Motor performance, **(B)** Visual search, **(C)** Working memory, **(D)** Vigilance, **(E)** Inhibition, **(F)** Domain-General.

Under a simple additive model, analysis of 253 effects across 42 studies in the motor domain revealed little consistency in the relationships between stimulation/experiment parameters and the likelihood of conferring positive or negative behavioral effects (Figure 2A; Table 3). However, there was a considerable advantage when using a within-subjects design vs. a between-subjects design, such that within-subjects designs were consistently associated with stronger positive effects (*d* = 1.35, 95CI = [0.93, 1.78]; *b* = 1.11 SD = 0.22, 95CI = [0.68, 1.55], *p*_d_ = 1). We also observed some evidence for target-specific effects, such that stimulating DLPFC tended to yield smaller behavioral differences, relative to the more motor-relevant cerebellum (*d* = −0.53, 95CI = [−1.13, 0.002]; *b* = −0.43, SD = 0.22, 95CI = [−0.86, −0.01], *p*_d_ = 0.977), although the credibility intervals for this effect lightly-intersect zero. We note that, due to restrictions on the number of possible contrasts one can uniquely-estimate for a given factor, there remain many potential comparisons that are not reflected under the model fit. However, given that this is a Bayesian framework, it is possible to ‘simulate’ new data under the generative model and derive *posterior predictive distributions* that describe the expected value (i.e., the likely effect size) one would observe under certain stimulation/experiment parameters. By integrating out other factors from these predictive distributions, we can approximate the *average marginal effect* (AME) one might expect for novel comparisons. This here reveals potential benefits to stimulating M1 vs. DLPFC (Median predictive difference = 0.35, 95CI = [0.11, 0.58]) as well as a potential detriment to stimulating online vs. offline (Median predictive difference = −0.20, 95CI = [−0.39, −0.01]).

**Table 3:** Summary of meta-regression parameters for motor performance.

Fixed predictors	Post. Med. Est.	Post. SD	95CI (*p*_d_)
Intercept	−0.04	0.62	−1.32 – 1.22 (0.524)
Polarity: Anodal – Cathodal	−0.07	0.08	−0.23 – 0.08 (0.829)
Target: DLPFC – Cerebellum	−0.43	0.22	−0.86 – −0.01 (0.977)
Target: M1 – Cerebellum	−0.09	0.19	−0.47 – 0.29 (0.672)
Target: Parietal – Cerebellum	−0.15	0.20	−0.55 – 0.25 (0.767)
Target: Other PFC – Cerebellum	−0.41	0.42	−1.24 – 0.41 (0.838)
Individualized target: Yes – No	−0.23	0.22	−0.68 – 0.19 (0.854)
Laterality: Left – Bilateral	−0.10	0.19	−0.48 – 0.26 (0.709)
Laterality: Right – Bilateral	−0.05	0.15	−0.34 – 0.25 (0.627)
Number of stimulation sessions	0.02	0.04	−0.06 – 0.12 (0.718)
Stimulation duration/session (min)	0.03	0.03	−0.04 – 0.10 (0.801)
Stimulation intensity (mA)	−0.16	0.26	−0.71 – 0.36 (0.725)
Stimulation timing: Offline – Mixed	0.26	0.25	−0.24 – 0.77 (0.844)
Stimulation timing: Online – Mixed	0.06	0.24	−0.42 – 0.55 (0.591)
Number of electrodes in montage	−0.08	0.07	−0.24 – 0.06 (0.863)
Design: Within – Between	1.11	0.22	0.68–1.55 (1.00)
**Random effects (*N* studies = 42)**			
σ_StudyID_ (Intercept)	0.82	0.14	0.58–1.14
*N* total effects = 253			
Marginal *R*^2^ = 0.25 [0.16, 0.34]			
Conditional *R*^2^ = 0.41 [0.34, 0.47]			

Applying the candidate interaction terms described previously to the motor domain did not reveal any significant increases in performance relative to the base model (Supplementary Table S5). The three-way interaction model including Design Type × Num Electrodes × Stim Duration provided the best overall predictive accuracy—however, this was not meaningfully different from the cross-validated performance of the base model with only additive terms (*ELPD_Diff_* = −33.53, *SE_Diff_* = 16.58, *ELPD_Diff_* / *SE_Diff_* = −1.88). That being said, while the highest-performing candidate model did not significantly outperform the base model, it nevertheless suggested the presence of potential interaction effects including (most importantly) the full three-way interaction (*b* = 0.76, SD = 0.28, 95CI = [0.21, 1.31], *p*_d_ = 0.996). It is unclear whether the increase in model complexity is justifiable given the nonsignificant difference in predictive performance, but we report the full set of parameter estimates for this model in Supplementary Table S6.

### 3.2 Visual search

The visual search model spanned 176 effects across 18 studies (Figure 2B; Table 4). One challenge of this domain was the lack of variability in stimulation target sites. The posterior parietal cortex (PPC) was highly overrepresented as a stimulation target in this sample, comprising 110/176 effects; conversely, only four effects were derived from visual cortex stimulation. The remaining regions spanned various areas of the prefrontal cortex, including the FEF (14 effects) and DLPFC (16 effects). We elected to retain the visual cortex target as its own factor level (although this estimate is highly unreliable, as reflected by the wide credibility interval) and collapse all PFC regions into one group, yielding 62 total effects. Bilateral stimulation (14/176 effects) and mixed online/offline stimulation designs (15/176 effects) were also underrepresented here.

**Table 4:** Summary of meta-regression parameters for visual search.

Fixed predictors	Post. Med. Est.	Post. SD	95CI (*p*_d_)
Intercept	0.25	0.98	−1.86 – 2.29 (0.599)
Polarity: Anodal – Cathodal	−0.22	0.08	−0.37 – −0.06 (0.996)
Target: PPC – PFC	−0.23	0.10	−0.42 – −0.03 (0.988)
Target: Visual – PFC	0.38	0.50	−0.67 – 1.40 (0.773)
Laterality: Left – Bilateral	−0.19	0.26	−0.70 – 0.31 (0.777)
Laterality: Right – Bilateral	−0.10	0.25	−0.59 – 0.38 (0.658)
Number of stimulation sessions	0.11	0.13	−0.16 – 0.38 (0.801)
Stimulation duration/session (min)	0.01	0.02	−0.02 – 0.05 (0.753)
Stimulation intensity (mA)	0.10	0.15	−0.20 – 0.38 (0.743)
Stimulation timing: Offline – Mixed	−0.32	0.23	−0.79 – 0.13 (0.920)
Stimulation timing: Online – Mixed	−0.25	0.17	−0.59 – 0.08 (0.928)
Number of electrodes in montage	−0.19	0.44	−1.11 – 0.76 (0.664)
Design: Within – Between	0.19	0.26	−0.36 – 0.74 (0.760)
**Random effects (*N* studies = 18)**			
σ_StudyID_ (Intercept)	0.40	0.11	0.23–0.65
*N* total effects = 176			
Marginal *R*^2^ = 0.36 [0.10, 0.49]			
Conditional *R*^2^ = 0.50 [0.39, 0.59]			

Despite the disparities across several of these parameters, we observed evidence for a moderate effect of stimulation polarity: cathodal stimulation was associated with smaller behavioral effects in these studies relative to anodal stimulation (*d* = −0.55, 95CI = [−1.14, −0.08]; *b* = −0.22, SD = 0.08, 95CI = [−0.37, −0.06], *p*_d_ = 0.996). Perhaps counterintuitively, we also saw evidence for moderately weaker behavioral effects when targeting PPC compared to the combined grouping of all PFC targets (*d* = −0.58, 95CI = [−1.29, −0.004]; *b* = −0.23, SD = 0.10, 95CI = [−0.42, −0.03], *p*_d_ = 0.988), although this effect is difficult to interpret given the lack of specificity in PFC regions. No additional trends were observed for other fixed effects or predictive AMEs in this domain.

With respect to potential interaction effects, here the base model provided the best predictive performance—strongly exceeding candidate interaction models with the exception of a model including a Design Type × Stim Intensity × Stim Duration interaction, with which it was tightly competitive (*ELPD_Diff_* = −0.10, *SE_Diff_* = 1.59, *ELPD_Diff_* / *SE_Diff_* = −0.06; Supplementary Table S7). Notably, however, despite the similarity in predictive performance, neither the three-way interaction term nor any of the marginal two-way interaction terms for this model provided clear evidence for nonzero effects. We observed, at best, an inconclusive trend for a two-way interaction between stimulation intensity and duration where the 95% credibility interval over the posterior included zero (*b* = 0.05, SD = 0.03, 95CI = [−0.003, 0.11], *p*_d_ = 0.969). Beyond this, we only observed evidence for an effect of stimulation polarity in the same direction as seen in the base model (i.e., such that cathodal stimulation was associated with reduced behavioral effects on average; *d* = −0.95, 95CI = [−3.24, −0.02]; *b* = −0.23, SD = 0.08, 95CI = [−0.39, −0.07], *p*_d_ = 0.998). The full summary of statistics for this competitive interaction model are compiled in Supplementary Table S8.

### 3.3 Working memory

We next assessed 115 effects reported in 16 working memory studies (Figure 2C; Table 5). Here we faced similar difficulties with unbalanced representation of target brain regions, with montages focused on the DLPFC in 90/115 reported effects. Other sparsely-represented regions (including the cerebellum, PPC, and temporal lobe) were therefore grouped into a ‘Non-DLPFC’ category. This domain was also the only other to employ subject-specific, individualized targeting—although this occurred only in one study with 5 reported effects. Furthermore, unlike other domains which applied anodal and cathodal stimulation fairly evenly, these studies almost exclusively applied anodal stimulation (103/115 effects). Bilateral stimulation also failed to be represented in this domain. Ultimately, none of the available contrasts suggested a reliable advantage or disadvantage to working memory performance, nor did examination of posterior predictive AMEs. Removal of highly-unbalanced factors also had no effect on the results, so we retain them here for transparency.

**Table 5:** Summary of meta-regression parameters for working memory.

Fixed predictors	Post. Med. Est.	Post. SD	95CI (*p*_d_)
Intercept	0.67	0.41	−0.20 – 1.58 (0.943)
Polarity: Anodal – Cathodal	0.08	0.13	−0.17 – 0.34 (0.725)
Target: Non-DLPFC – DLPFC	−0.06	0.10	−0.25 – 0.13 (0.734)
Individualized target: Yes – No	0.65	0.35	−0.08 – 1.40 (0.963)
Laterality: Right – Left	0.03	0.10	−0.16 – 0.22 (0.605)
Number of stimulation sessions	0.02	0.04	−0.07 – 0.10 (0.692)
Stimulation duration/session (min)	0.01	0.01	−0.01 – 0.04 (0.833)
Stimulation intensity (mA)	−0.44	0.26	−1.01 – 0.09 (0.953)
Stimulation timing: Offline – Mixed	0.35	0.21	−0.11 – 0.81 (0.941)
Stimulation timing: Online – Mixed	0.26	0.19	−0.16 – 0.69 (0.906)
Number of electrodes in montage	0.10	0.07	−0.05 – 0.25 (0.921)
Design: Within – Between	−0.14	0.18	−0.54 – 0.23 (0.786)
**Random effects (*N* studies = 16)**			
σ_StudyID_ (Intercept)	0.22	0.11	0.04–0.47
*N* total effects = 115			
Marginal *R*^2^ = 0.16 [0.06, 0.31]			
Conditional *R*^2^ = 0.14 [0.07, 0.21]			

Turning to our model comparison set, the best performing model included a three-way interaction between Design Type × Stim Intensity × Stim Duration—but again, this failed to significantly beat the base model in terms of predictive performance (*ELPD_Diff_* = −3.10, *SE_Diff_* = 2.50, *ELPD_Diff_* / *SE_Diff_* = −1.24; Supplementary Table S9). Furthermore, this particular candidate model, while ostensibly offering the highest predictive utility, also demonstrated more degenerate behavior during sampling, consistently yielding several divergent Monte Carlo transitions regardless of numerous attempts at adjusting sampling behavior. While these make up an extremely small proportion of the total number of MCMC iterations run, it nevertheless suggests a more unwieldy posterior geometry, and this in turn can hamper the interpretability/reliability of parameter estimates. Here we see several potential effects that were not apparent in the base model, including a potential advantage for using larger electrode montages (*b* = 0.14, SD = 0.06, 95CI = [0.02, 0.27], *p*_d_ = 0.989) and some evidence trending towards a two-way interaction between stimulation intensity and duration (*b* = −0.15, SD = 0.05, 95CI = [−0.25, −0.04], *p*_d_ = 0.991). The full set of parameter estimates for this model can be found in Supplementary Table S10, although we urge some caution in their interpretation given the aforementioned challenges with convergence.

### 3.4 Vigilance

The effects of tDCS on performance in vigilance tasks were modeled over 15 studies reporting 93 effects (Figure 2D; Table 6). Similar to working memory, most studies here employed anodal stimulation designs (83 effects) focused on the DLPFC (51 effects). Additional brain regions were grouped into ‘Other PFC’ and ‘Temporal–Parietal’ categories. Most design parameters did not suggest any associations with behavioral outcomes; however, we observed a small positive relationship with the number of electrodes used in the montage (*b* = 0.08, SD = 0.04, 95CI = [0.01, 0.15], *p*_d_ = 0.983). We note that this may have been driven by two studies using a more complex montage comprised of 10 electrodes (yielding 11 effects)—other studies employed between 2 and 5 electrodes. Thus, we caution against overinterpreting this particular effect. Further comparisons of posterior predictive densities and AMEs did not reveal additional associations beyond the estimated contrasts.

**Table 6:** Summary of meta-regression parameters for vigilance.

Fixed predictors	Post. Med. Est.	Post. SD	95CI (*p*_d_)
Intercept	−0.81	0.67	−2.19 – 0.57 (0.883)
Polarity: Anodal – Cathodal	0.03	0.12	−0.20 – 0.26 (0.607)
Target: Other PFC – DLPFC	0.03	0.23	−0.48 – 0.50 (0.552)
Target: Temporal–Parietal – DLPFC	−0.00	0.09	−0.19 – 0.18 (0.526)
Laterality: Left – Bilateral	0.10	0.21	−0.35 – 0.55 (0.687)
Laterality: Right – Bilateral	0.07	0.26	−0.50 – 0.58 (0.601)
Number of stimulation sessions	0.01	0.17	−0.35 – 0.37 (0.528)
Stimulation duration/session (min)	0.02	0.02	−0.03 – 0.08 (0.841)
Stimulation intensity (mA)	0.00	0.22	−0.49 – 0.46 (0.507)
Stimulation timing: Offline – Mixed	−0.04	0.16	−0.38 – 0.28 (0.595)
Stimulation timing: Online – Mixed	−0.01	0.13	−0.28 – 0.25 (0.536)
Number of electrodes in montage	0.08	0.04	0.01–0.15 (0.982)
Design: Within – Between	0.12	0.09	−0.07 – 0.29 (0.891)
**Random effects (*N* studies = 15)**			
σ_StudyID_ (Intercept)	0.13	0.09	0.01–0.35
*N* total effects = 93			
Marginal *R*^2^ = 0.40 [0.23, 0.53]			
Conditional *R*^2^ = 0.41 [0.26, 0.52]			

The base model further demonstrated the best performance relative to all candidate interaction models; however, it was not *significantly better* than any candidate model. The closest contender was again the model including a Design Type × Stim Intensity × Stim Duration interaction (*ELPD_Diff_* = −0.67, *SE_Diff_* = 1.48, *ELPD_Diff_* / *SE_Diff_* = −0.45; Supplementary Table S11)—although no individual main effects or interaction effects under this model yielded any nonzero effects (Supplementary Table S12). Thus, the inclusion of interaction terms does not inform differences under this domain any more than those scantly observed in the base model.

### 3.5 Inhibition

A survey of 84 effects across 15 inhibition studies did not reveal any meaningful associations between any stimulation or experiment parameters and behavior (Figure 2E; Table 7). Again, there was little variability in brain regions targeted for stimulation: 75/84 effects were localized to the DLPFC. The remaining effects spanned the inferior frontal junction and parietal cortex and were therefore collapsed into an ‘Other’ term during the original model fit. Removing this predictor from the model did not affect the results. There was also less variability in target laterality (no effects under bilateral stimulation) and the number of stimulation sessions (only one session reported per subject). Assessments of posterior predictive densities and AMEs did not reveal any other potential effects not captured in the base model. Although several parameters trended towards a nonzero effect, there is inconclusive evidence for any reliable differences in behavioral performance given variation in design parameters under this domain.

**Table 7:** Summary of meta-regression parameters for inhibition.

Fixed predictors	Post. Med. Est.	Post. SD	95CI (*p*_d_)
Intercept	0.71	0.62	−0.53 – 2.14 (0.879)
Polarity: Anodal – Cathodal	−0.09	0.08	−0.24 – 0.07 (0.865)
Target: Other – DLPFC	0.17	0.14	−0.10 – 0.45 (0.892)
Laterality: Right – Left	−0.12	0.12	−0.36 – 0.12 (0.836)
Stimulation duration/session (min)	−0.01	0.02	−0.06 – 0.03 (0.751)
Stimulation intensity (mA)	0.00	0.14	−0.31 – 0.29 (0.514)
Stimulation timing: Offline – Mixed	0.19	0.30	−0.39 – 0.90 (0.745)
Stimulation timing: Online – Mixed	−0.03	0.33	−0.65 – 0.73 (0.534)
Number of electrodes in montage	−0.10	0.08	−0.29 – 0.07 (0.900)
Design: Within – Between	0.04	0.21	−0.40 – 0.53 (0.581)
**Random effects (*N* studies = 15)**			
σ_StudyID_ (Intercept)	0.35	0.14	0.14–0.68
*N* total effects = 84			
Marginal *R*^2^ = 0.23 [0.09, 0.40]			
Conditional *R*^2^ = 0.36 [0.22, 0.50]			

Here we again saw superior performance for the base model over candidate interaction models. This time, however, the base model consistently, significantly beat all three potential candidates—no exploratory model provided competitive predictive performance (Supplementary Table S13).

### 3.6 Combined model

Finally, we considered a domain-general model combining all 106 studies and 721 effects across all domains (Figure 2F; Table 8). Here we omitted the individualized target parameter (due to its scarce representation beyond motor performance tasks) as well as the regional target parameter to instead focus on broader trends related to stimulation and experiment design. Taken together, there was considerable heterogeneity across all relevant effects, with stimulation polarity and within- vs. between-subject designs providing inconclusive evidence for domain-general associations with behavior: cathodal stimulation tended to produce somewhat smaller behavioral effects on average (*d* = −0.19, 95CI = [−0.39, 0.02]; *b* = −0.07, SD = 0.04, 95CI = [−0.15, 0], *p*_d_ = 0.971), while within-subjects designs produced larger effects (*d* = 0.32, 95CI = [0, 0.65]; *b* = 0.13, SD = 0.07, 95CI = [0, 0.26], *p*_d_ = 0.973). Posterior predictive AMEs further suggested that online stimulation may yield smaller effects than offline stimulation (Median predictive difference = −0.14, 95CI = [−0.24, −0.04]).

**Table 8:** Summary of meta-regression parameters across all domains.

Fixed predictors	Post. Med. Est.	Post. SD	95CI (*p*_d_)
Intercept	−0.01	0.15	−0.31 – 0.29 (0.532)
Polarity: Anodal – Cathodal	−0.07	0.04	−0.15 – 0.00 (0.971)
Laterality: Left – Bilateral	−0.13	0.09	−0.31 – 0.06 (0.914)
Laterality: Right – Bilateral	−0.12	0.09	−0.30 – 0.06 (0.904)
Number of stimulation sessions	0.01	0.02	−0.03 – 0.05 (0.660)
Stimulation duration/session (min)	0.01	0.01	−0.01 – 0.02 (0.866)
Stimulation intensity (mA)	0.02	0.07	−0.11 – 0.15 (0.619)
Stimulation timing: Offline – Mixed	0.03	0.09	−0.15 – 0.21 (0.624)
Stimulation timing: Online – Mixed	−0.11	0.09	−0.28 – 0.06 (0.893)
Number of electrodes in montage	0.01	0.02	−0.04 – 0.06 (0.681)
Design: Within – Between	0.13	0.07	0.00–0.26 (0.972)
**Random effects (*N* studies = 106)**			
σ_StudyID_ (Intercept)	0.39	0.04	0.32–0.46
*N* total effects = 721			
Marginal *R*^2^ = 0.05 [0.02, 0.09]			
Conditional *R*^2^ = 0.29 [0.25, 0.33]			

As described previously, the competing set of three-way interaction models was originally defined by concatenating together the data from all domains. Thus, each candidate model significantly outperformed the base model (Supplementary Table S4) while remaining competitive with each other (Supplementary Table S14). The best-performing candidate included a strong three-way interaction between Design Type × Num Electrodes × Stim Intensity (*b* = −0.49, SD = 0.10, 95CI = [−0.69, −0.29], *p*_d_ = 1), which suggested that the montages with more electrodes may produce larger behavioral effects as raw stimulation intensity increases under *between-subjects* designs—but this relationship is reversed in *within-subjects* designs, such that one might expect increasingly-diminishing returns with more complex montages and stronger stimulation intensities (Supplementary Figure S2; Supplementary Table S15). The second-best model included a three-way interaction for Design Type × Stim Intensity × Stim Duration (*b* = −0.11, SD = 0.03, 95CI = [−0.17, −0.06], *p*_d_ = 1): here, we saw similar trends as in the previous model, such that longer and stronger applications of tDCS may confer an advantage in between-subjects studies, but this is again reversed for within-subjects designs (Supplementary Figure S3; Supplementary Table S16). Finally, the third candidate model provided evidence for a three-way interaction between Design Type × Num Electrodes × Stim Duration (*b* = 0.06, SD = 0.02, 95CI = [0.03, 0.09], *p*_d_ = 1), where longer stimulation durations coupled with larger montages may provide a specific benefit to within-subject designs, whereas between-subject designs are relatively invariant to combined changes in these factors (Supplementary Figure S4; Supplementary Table S17).

## 4 Discussion

When designing a neurostimulation experiment, researchers are presented with a vast decision tree. For example, one must determine where in the brain to apply stimulation; to what intensity and for how long; whether to apply it *online* (as subjects are performing a task) or *offline*; and whether to apply ‘excitatory’ or ‘inhibitory’ stimulation. The net effect of neurostimulation ultimately depends on a complex constellation of these factors—and while some of these decisions have clear theoretical/empirical guides (e.g., targeting brain regions previously known to be associated with task performance), many others remain open to the subjective preferences of the researcher, and there is no clear consensus or standardization of the optimal approach. In this report, we sought to identify which of these stimulation and experimental design factors confer the strongest likelihood of behavioral effects in tDCS studies, using a hierarchical, Bayesian meta-regression approach across five cognitive domains.

We observed considerable heterogeneity in these associations, with little consensus between domains. When combining all studies and effects under a purely-additive, domain-general model, only two factors emerged with a modicum of consistency: (1) the polarity of stimulation, with cathodal vs. sham designs often producing reduced behavioral effects relative to anodal vs. sham designs (perhaps in line with the naïve notion that cathodal stimulation is primarily *inhibitory*), and (2) within-subject experimental designs conferring larger effects than between-subject designs (which may be unsurprising given that within-subject comparisons are generally considered to be more powerful—which is to say, for an identical assumed effect size *a priori*, within-subject manipulations require fewer subjects to reach the same level of power relative to between-subjects). These associations were modest at best when considering the full range of variance in reported effects; however, stronger associations with polarity and statistical design were seen across visual search and motor performance tasks, respectively.

The results of our exploratory interaction analyses are also somewhat difficult to interpret, given that no candidate model significantly generalized to individual cognitive domains—the improvements in predictive performance when including various three-way interaction terms were most appreciable when combining data across domains. However, there are several notable trends that may be worthy of consideration. For example, the use of between- vs. within-subject designs was consistently a relevant factor in each candidate model. In cases where this factor interacted with Stimulation Intensity × Num Electrodes (Supplementary Figure S2; Supplementary Table S15) and Stimulation Intensity × Stim Duration (Supplementary Figure S3; Supplementary Table S16), it appeared that the benefit of larger montages and longer stimulation durations (respectively) reversed for between- vs. within-subject designs—however, this switch primarily occurred out in the tails of the observed stimulation intensities (i.e., at least 3 mA or more). While such high intensities were seen in our review, they are *not* necessarily representative of most tDCS studies on the whole. At lower, more traditional stimulation intensities (e.g., ~1 mA), within-subjects designs generally still held an advantage over between-subjects. Furthermore, it is worth noting that factors such as stimulation intensity generally only informed differences in behavioral outcomes when included in an interaction—this seems reasonable given that the effectiveness of a given intensity may critically depend upon the duration of stimulation and/or the extent of an electrode montage. However, deeper interpretation of these results is made somewhat difficult given that raw intensity alone does not necessarily reflect the *dosage* of stimulation applied to the target (typically operationalized as current density), nor does the three-way interaction identified here. We attempted to model this factor in each domain (Supplementary Figure S1), but it was not reliably estimable for all studies (resulting in loss of data) and otherwise did not appear to be predictive of outcome variability. There are a number of potential reasons for this (e.g., individual differences in brain anatomy mediating differences in the effective current density at the target) which warrant future investigation, and it is similarly critical for other empirical work to report this information in their Methods sections to help aid in the interpretation of results. In sum, while it nevertheless remains unclear as to whether (or to what extent) specific stimulation or experimental parameters can reliably induce desired behavioral outcomes, these findings may provide a useful reference for researchers designing future behavioral studies in addition to more systematic investigations of how various tDCS parameters may interact to modulate behavior.

A growing number of published meta-analyses have attempted to quantify and characterize patterns of uncertainty and variability in neurostimulation-related effects on cognition and behavior. However, to date, many of these previous efforts have taken a more piecemeal approach, focused on specific tasks/domains, individual behavioral measures (e.g., accuracy or reaction time), or a limited range of stimulation parameters (e.g., particular target brain regions, or differentiating between anodal or cathodal stimulation alone) (Jacobson et al., 2012; Brunoni and Vanderhasselt, 2014; Dedoncker et al., 2016; Hill et al., 2016; Mancuso et al., 2016; Imburgio and Orr, 2018; Oldrati and Schutter, 2018; Schroeder et al., 2020). Our goal, by contrast, was to take a more holistic view capturing a broad range of design factors and behaviors. This presented a number of challenges, including uneven representation of some stimulation/experimental parameters across domains, making it difficult to robustly contrast certain effects. In a sense, these imbalances could be interpreted as a *de facto* consensus on which brain regions ‘should’ be targeted, or which type of stimulation to apply, etc., when probing behaviors under a given domain. Yet, our analyses demonstrate that there is still a profound degree of heterogeneity across these parameters, suggesting the need for deeper consideration when planning and designing tDCS studies.

### 4.1 Uncontrollable sources of variability

The design elements under investigation here are generally considered to be *controllable* sources of variability in neurostimulation studies (i.e., easily-manipulated by experimenters in a systematic fashion), but there nevertheless remain a host of *uncontrollable* sources of variability that may further hamper one’s ability to elicit behavioral effects and contribute to inconsistent results across studies. Critically among these are a number of neuroanatomical factors that likely influence the propagation of electrical current through the brain and the degree to which stimulation actually produces the presumed effects on neuronal activity. For example, neurons running parallel to an applied electric field generally appear to exhibit the hypothesized excitatory and inhibitory effects of anodal and cathodal stimulation (respectively), but neurons running orthogonally to the field may not (Rahman et al., 2013; Lafon et al., 2017). Inter-individual variability in the local orientation of neurons within a target brain region might therefore preclude effective neuromodulation during tDCS. Other variables such as head size, head fat content, skull thickness, and sulcal depth can also mediate the effectiveness of tDCS (Kessler et al., 2013; Truong et al., 2013; Opitz et al., 2015; Bikson et al., 2016), which together could manifest as further individual differences in behavior, washing out a mean difference between one stimulation/task condition and another.

The development of improved, neuroanatomically-informed current modeling techniques, particularly with respect to individual-specific optimization of electrode placements and stimulation dosage, may be able to mitigate some of these factors (Datta et al., 2012; Kim et al., 2014; Woods et al., 2016; Saturnino et al., 2019). Recent advances in electrical field modeling, including the Realistic Volumetric Approach to Simulate Transcranial Electrical Stimulation (ROAST) software (Huang et al., 2019), the dose-target determination index (DTDI) (Kashyap et al., 2021), and other novel approaches combining meta-analysis with simulation of electrical fields (Wischnewski et al., 2021) have highlighted that it is not sufficient to simply place an electrode montage over a given brain area and assume that it is receiving focal stimulation. The frequently-observed mixed findings across tDCS studies may in large part be due to variability and suboptimality in the actual strength/focality of electric fields over the desired target brain region (Wischnewski et al., 2021). We advocate strongly for the use of these field modeling techniques whenever possible as an additional control over stimulation targeting.

Finally, and more generally, there remain many other challenging sources of variability (e.g., baseline cognitive abilities, neurochemistry, circadian rhythms, blood pressure, genetic factors, etc.) (Li et al., 2015; Hsu et al., 2016; Rosen et al., 2016) which, while not necessarily *impossible* to control for, are frequently impractical or unrealistic for any given study to measure and factor into their analyses. Because these variables are often so difficult to quantify (and as such, are rarely reported), the sum total of their influence on the heterogenous nature of effects modeled here cannot be ascertained. Nevertheless, a more complete understanding of these issues is imperative as the field moves forward.

### 4.2 Limitations of this study

As aforementioned, we applied highly-restrictive inclusion criteria when collating data for this meta-analysis. While this was necessary to enable more straightforward tests of our key stimulation and experimental parameters, these findings may not be fully-representative of the literature on the whole (although the high degree of variability observed is in line with prior work). One critical omission of note spans studies using customized/individualized stimulation protocols beyond target placement alone: for example, paradigms which vary stimulation intensity on an individual basis to standardize the dosage (current density) applied to the target. Our models required stimulation intensity to be held constant for a given reported effect, but such individualized approaches are becoming increasingly more commonplace. Thus, while our results may reflect historical trends in stimulation outcomes (especially with respect to conventional designs), they may not adequately account for present trends using more sophisticated methods. Future meta-analytic work would benefit from more direct comparisons between conventional and contemporary approaches to stimulation designs.

As an additional limitation, we desired to identify more generalizable trends in how these design parameters relate to behavioral outcomes, both within and between domains—but collapsing across multiple task paradigms and performance metrics may actually obscure reliable effects that would otherwise be apparent in a more focal meta-analysis. Even when simplifying behavioral effects to ‘positive’ and ‘negative’ outcomes, it is unlikely that any given study tests/measures a particular psychological construct in the same way, which might amplify the inherent noise variance under the model.

Finally, while we attempted to explore potential interactions between various experiment design factors, this was still somewhat limited in scope—to limit complexity and reduce the putative model space, we considered at most the inclusion of a single three-way interaction term (along with its marginal two-way interactions) and performed this selection based on data combined across domains rather than within each domain. While on the one hand this allowed us to assess generalizability of the identified candidate models, it is also possible that each domain may produce different sets of candidate interactions. Furthermore, in order to be consistent with our base, additive models across all domains, we retained the same sets of weaky-informative priors: but in a Bayesian model comparison and variable selection scenario, sparsity-inducing priors (e.g., a regularized horseshoe) may be preferable. These are often extremely delicate in the context of hierarchical models, though, and may produce degenerate sampling behavior and difficulties with convergence. Future experimental work would greatly benefit from systematic investigations of these parameters and the potential ways in which they jointly-modulate behavioral performance outcomes, and this modeling framework could also potentially be combined with tDCS simulation methods (Wischnewski et al., 2021) to further inform experimental design principles for neurostimulation researchers.

## 5 Conclusion

In conclusion, our comprehensive meta-analysis across five cognitive domains reveals a complex and heterogenous landscape of associations between neurostimulation and behavioral outcomes. While some trends emerge, such as the advantage of within-subject designs and the influence of stimulation polarity, overall, there is little consensus on the optimal stimulation and experimental parameters for reliably inducing desired behavioral outcomes using tDCS. The field of neurostimulation research faces the challenge of contending with uncontrollable sources of variability, including various neuroanatomical and individual difference factors, which may further contribute to the inconsistency observed across studies. This study underscores the need for deeper consideration and standardization of experimental design in tDCS studies and highlights the importance of developing neuroanatomically-informed current modeling techniques to better understand and control for sources of variability. Future research should continue to investigate the complex interplay of these parameters and their potential interactions to advance our understanding of the effects of tDCS on cognitive and behavioral performance.

## Data availability statement

The raw data supporting the conclusions of this article will be made available by the authors, without undue reservation.

## Author contributions

TS: Conceptualization, Data curation, Formal analysis, Methodology, Writing – original draft, Writing – review & editing. SL: Conceptualization, Data curation, Writing – review & editing. LL: Conceptualization, Data curation, Writing – review & editing. HS: Data curation, Writing – review & editing. JS: Data curation, Writing – review & editing. PS: Data curation, Writing – review & editing. KD: Data curation, Writing – review & editing. MM: Conceptualization, Funding acquisition, Project administration, Supervision, Writing – review & editing. TB: Conceptualization, Funding acquisition, Project administration, Supervision, Writing – original draft, Writing – review & editing.

## Footnotes

^1^ http://tdcsdatabase.com/

^2^ https://automeris.io/WebPlotDigitizer/

^3^ https://mc-stan.org
