# Supplementary material for: Towards optimized methodological parameters for maximizing the behavioral effects of transcranial direct current stimulation
Source: Front Hum Neurosci. 2024 Jul 2;18:1305446. doi: 10.3389/fnhum.2024.1305446 (PMC11250584; doi:10.3389/fnhum.2024.1305446)
Supplement: Supplementary file 1 [file Data_Sheet_1.docx]

**Supplementary Information**

Towards Optimized Methodological Parameters for Maximizing

the Behavioral Effects of Transcranial Direct Current Stimulation

Tyler Santander, Sara Leslie, Luna J. Li, Henri E. Skinner, Jessica M. Simonson, Patrick Sweeney, Kaitlyn P. Deen, Michael B. Miller, & Tad T. Brunye

**Table S1.** Studies included under each cognitive domain with reference numbers corresponding to their appearance in the full text.

| **Motor Performance** | | |
| --- | --- | --- |
| **Ref no.** | **Citation** | |
| 112 | Apsvalka D, Ramsey R, Cross ES. Anodal tDCS over Primary Motor Cortex Provides No Advantage to Learning Motor Sequences via Observation. Neural Plast. 2018;2018:1237962. | |
| 113 | Antal A, Nitsche MA, Kincses TZ, Kruse W, Hoffmann KP, Paulus W. Facilitation of visuo-motor learning by transcranial direct current stimulation of the motor and extrastriate visual areas in humans. Eur J Neurosci. 2004;19(10):2888-92. | |
| 114 | Ambrus GG, Chaieb L, Stilling R, Rothkegel H, Antal A, Paulus W. Monitoring transcranial direct current stimulation induced changes in cortical excitability during the serial reaction time task. Neurosci Lett. 2016;616:98-104. | |
| 115 | Zimerman M, Heise KF, Gerloff C, Cohen LG, Hummel FC. Disrupting the ipsilateral motor cortex interferes with training of a complex motor task in older adults. Cereb Cortex. 2014;24(4):1030-6. | |
| 116 | von Rein E, Hoff M, Kaminski E, Sehm B, Steele CJ, Villringer A, et al. Improving motor performance without training: the effect of combining mirror visual feedback with transcranial direct current stimulation. J Neurophysiol. 2015;113(7):2383-9. | |
| 117 | Vollmann H, Conde V, Sewerin S, Taubert M, Sehm B, Witte OW, et al. Anodal transcranial direct current stimulation (tDCS) over supplementary motor area (SMA) but not pre-SMA promotes short-term visuomotor learning. Brain Stimul. 2013;6(2):101-7. | |
| 118 | Vines BW, Cerruti C, Schlaug G. Dual-hemisphere tDCS facilitates greater improvements for healthy subjects' non-dominant hand compared to uni-hemisphere stimulation. BMC Neurosci. 2008;9:103. | |
| 119 | Toth AJ, Ramsbottom N, Constantin C, Milliet A, Campbell MJ. The effect of expertise, training and neurostimulation on sensory-motor skill in esports. Computers in Human Behavior. 2021;121:106782. | |
| 120 | Tecchio F, Zappasodi F, Assenza G, Tombini M, Vollaro S, Barbati G, et al. Anodal transcranial direct current stimulation enhances procedural consolidation. J Neurophysiol. 2010;104(2):1134-40. | |
| 121 | Talimkhani A, Abdollahi I, Mohseni-Bandpei MA, Ehsani F, Khalili S, Jaberzadeh S. Differential Effects of Unihemispheric Concurrent Dual-Site and Conventional tDCS on Motor Learning: A Randomized, Sham-Controlled Study. Basic Clin Neurosci. 2019;10(1):59-72. | |
| 122 | Spampinato DA, Satar Z, Rothwell JC. Combining reward and M1 transcranial direct current stimulation enhances the retention of newly learnt sensorimotor mappings. Brain Stimul. 2019;12(5):1205-12. | |
| 123 | Shilo G, Lavidor M. Non-linear effects of cathodal transcranial direct current stimulation (tDCS) of the primary motor cortex on implicit motor learning. Exp Brain Res. 2019;237(4):919-25. | |
| 124 | Sevilla-Sanchez M, Hortobágyi T, Fogelson N, Iglesias-Soler E, Carballeira E, Fernandez-del-Olmo M. Small Enhancement of Bimanual Typing Performance after 20 Sessions of tDCS in Healthy Young Adults. Neuroscience. 2021;466:26-35. | |
| 125 | Saucedo Marquez CM, Zhang X, Swinnen SP, Meesen R, Wenderoth N. Task-specific effect of transcranial direct current stimulation on motor learning. Front Hum Neurosci. 2013;7:333. | |
| 126 | Samaei A, Ehsani F, Zoghi M, Hafez Yosephi M, Jaberzadeh S. Online and offline effects of cerebellar transcranial direct current stimulation on motor learning in healthy older adults: a randomized double-blind sham-controlled study. Eur J Neurosci. 2017;45(9):1177-85. | |
| 127 | Rumpf JJ, Wegscheider M, Hinselmann K, Fricke C, King BR, Weise D, et al. Enhancement of motor consolidation by post-training transcranial direct current stimulation in older people. Neurobiol Aging. 2017;49:1-8. | |
| 128 | Rocha K, Marinho V, Magalhaes F, Carvalho V, Fernandes T, Ayres M, et al. Unskilled shooters improve both accuracy and grouping shot having as reference skilled shooters cortical area: An EEG and tDCS study. Physiol Behav. 2020;224:113036. | |
| 129 | Prichard G, Weiller C, Fritsch B, Reis J. Effects of different electrical brain stimulation protocols on subcomponents of motor skill learning. Brain Stimul. 2014;7(4):532-40. | |
| 130 | Pollok B, Keitel A, Foerster M, Moshiri G, Otto K, Krause V. The posterior parietal cortex mediates early offline-rather than online-motor sequence learning. Neuropsychologia. 2020;146:107555. | |
| 131 | Pixa NH, Steinberg F, Doppelmayr M. High-definition transcranial direct current stimulation to both primary motor cortices improves unimanual and bimanual dexterity. Neurosci Lett. 2017;643:84-8. | |
| 132 | Parma JO, Profeta V, Andrade AGP, Lage GM, Apolinario-Souza T. TDCS of the Primary Motor Cortex: Learning the Absolute Dimension of a Complex Motor Task. J Mot Behav. 2021;53(4):431-44. | |
| 133 | Nguemeni C, Stiehl A, Hiew S, Zeller D. No Impact of Cerebellar Anodal Transcranial Direct Current Stimulation at Three Different Timings on Motor Learning in a Sequential Finger-Tapping Task. Front Hum Neurosci. 2021;15:631517. | |
| 134 | Naros G, Geyer M, Koch S, Mayr L, Ellinger T, Grimm F, et al. Enhanced motor learning with bilateral transcranial direct current stimulation: Impact of polarity or current flow direction? Clin Neurophysiol. 2016;127(4):2119-26. | |
| 135 | Minarik T, Sauseng P, Dunne L, Berger B, Sterr A. Effects of anodal transcranial direct current stimulation on visually guided learning of grip force control. Biology (Basel). 2015;4(1):173-86. | |
| 136 | Leite J, Carvalho S, Fregni F, Goncalves OF. Task-specific effects of tDCS-induced cortical excitability changes on cognitive and motor sequence set shifting performance. PLoS One. 2011;6(9):e24140. | |
| 137 | Jin Y, Lee J, Kim S, Yoon B. Noninvasive brain stimulation over M1 and DLPFC cortex enhances the learning of bimanual isometric force control. Hum Mov Sci. 2019;66:73-83. | |
| 138 | Hashemirad F, Fitzgerald PB, Zoghi M, Jaberzadeh S. Single-Session Anodal tDCS with Small-Size Stimulating Electrodes Over Frontoparietal Superficial Sites Does Not Affect Motor Sequence Learning. Front Hum Neurosci. 2017;11:153. | |
| 139 | Furuya S, Klaus M, Nitsche MA, Paulus W, Altenmuller E. Ceiling effects prevent further improvement of transcranial stimulation in skilled musicians. J Neurosci. 2014;34(41):13834-9. | |
| 140 | Fujiyama H, Hinder MR, Barzideh A, Van de Vijver C, Badache AC, Manrique CM, et al. Preconditioning tDCS facilitates subsequent tDCS effect on skill acquisition in older adults. Neurobiol Aging. 2017;51:31-42. | |
| 141 | Focke J, Kemmet S, Krause V, Keitel A, Pollok B. Cathodal transcranial direct current stimulation (tDCS) applied to the left premotor cortex (PMC) stabilizes a newly learned motor sequence. Behav Brain Res. 2017;316:87-93. | |
| 142 | Filmer HL, Lyons M, Mattingley JB, Dux PE. Anodal tDCS applied during multitasking training leads to transferable performance gains. Sci Rep. 2017;7(1):12988. | |
| 143 | Ferrucci R, Brunoni AR, Parazzini M, Vergari M, Rossi E, Fumagalli M, et al. Modulating human procedural learning by cerebellar transcranial direct current stimulation. Cerebellum. 2013;12(4):485-92. | |
| 144 | Fehring DJ, Illipparampil R, Acevedo N, Jaberzadeh S, Fitzgerald PB, Mansouri FA. Interaction of task-related learning and transcranial direct current stimulation of the prefrontal cortex in modulating executive functions. Neuropsychologia. 2019;131:148-59. | |
| 145 | Fan J, Voisin J, Milot MH, Higgins J, Boudrias MH. Transcranial direct current stimulation over multiple days enhances motor performance of a grip task. Ann Phys Rehabil Med. 2017;60(5):329-33. | |
| 146 | Ehsani F, Bakhtiary AH, Jaberzadeh S, Talimkhani A, Hajihasani A. Differential effects of primary motor cortex and cerebellar transcranial direct current stimulation on motor learning in healthy individuals: A randomized double-blind sham-controlled study. Neurosci Res. 2016;112:10-9. | |
| 147 | Dumel G, Bourassa ME, Charlebois-Plante C, Desjardins M, Doyon J, Saint-Amour D, et al. Multisession anodal transcranial direct current stimulation induces motor cortex plasticity enhancement and motor learning generalization in an aging population. Clin Neurophysiol. 2018;129(2):494-502. | |
| 148 | Dumel G, Bourassa ME, Charlebois-Plante C, Desjardins M, Doyon J, Saint-Amour D, et al. Motor Learning Improvement Remains 3 Months After a Multisession Anodal tDCS Intervention in an Aging Population. Front Aging Neurosci. 2018;10:335. | |
| 149 | Doppelmayr M, Pixa NH, Steinberg F. Cerebellar, but not Motor or Parietal, High-Density Anodal Transcranial Direct Current Stimulation Facilitates Motor Adaptation. J Int Neuropsychol Soc. 2016;22(9):928-36. | |
| 150 | Cantarero G, Spampinato D, Reis J, Ajagbe L, Thompson T, Kulkarni K, et al. Cerebellar direct current stimulation enhances on-line motor skill acquisition through an effect on accuracy. J Neurosci. 2015;35(7):3285-90. | |
| 151 | Boggio PS, Castro LO, Savagim EA, Braite R, Cruz VC, Rocha RR, et al. Enhancement of non-dominant hand motor function by anodal transcranial direct current stimulation. Neurosci Lett. 2006;404(1-2):232-6. | |
| 152 | Ballard HK, Eakin SM, Maldonado T, Bernard JA. Using high-definition transcranial direct current stimulation to investigate the role of the dorsolateral prefrontal cortex in explicit sequence learning. PLoS One. 2021;16(3):e0246849. | |
| 153 | Ballard HK, Goen JRM, Maldonado T, Bernard JA. Effects of cerebellar transcranial direct current stimulation on the cognitive stage of sequence learning. J Neurophysiol. 2019;122(2):490-9. | |
| **Visual Search** | | |
| **Ref no.** | **Citation** | |
| 88 | Nelson JM, McKinley RA, McIntire LK, Goodyear C, Walters C. Augmenting visual search performance with transcranial direct current stimulation (tDCS). Military Psychology. 2015;27(6):335-47. | |
| 89 | Ball K, Lane AR, Smith DT, Ellison A. Site-dependent effects of tDCS uncover dissociations in the communication network underlying the processing of visual search. Brain Stimul. 2013;6(6):959-65. | |
| 90 | Callan DE, Falcone B, Wada A, Parasuraman R. Simultaneous tDCS-fMRI Identifies Resting State Networks Correlated with Visual Search Enhancement. Front Hum Neurosci. 2016;10:72. | |
| 142 | Filmer HL, Lyons M, Mattingley JB, Dux PE. Anodal tDCS applied during multitasking training leads to transferable performance gains. Sci Rep. 2017;7(1):12988. | |
| 154 | Nydam AS, Sewell DK, Dux PE. Cathodal electrical stimulation of frontoparietal cortex disrupts statistical learning of visual configural information. Cortex. 2018;99:187-99. | |
| 155 | Cosman JD, Atreya PV, Woodman GF. Transient reduction of visual distraction following electrical stimulation of the prefrontal cortex. Cognition. 2015;145:73-6. | |
| 156 | Sung K, Gordon B. Transcranial direct current stimulation (tDCS) facilitates overall visual search response times but does not interact with visual search task factors. PLoS One. 2018;13(3):e0194640. | |
| 157 | Clark VP, Coffman BA, Mayer AR, Weisend MP, Lane TD, Calhoun VD, et al. TDCS guided using fMRI significantly accelerates learning to identify concealed objects. Neuroimage. 2012;59(1):117-28. | |
| 158 | Lanina AA, Feurra M, Gorbunova ES. No Effect of the Right Posterior Parietal Cortex tDCS in Dual-Target Visual Search. Front Psychol. 2018;9:2112. | |
| 159 | Falcone B, Wada A, Parasuraman R, Callan DE. Individual differences in learning correlate with modulation of brain activity induced by transcranial direct current stimulation. PLoS One. 2018;13(5):e0197192. | |
| 160 | Filmer HL, Varghese E, Hawkins GE, Mattingley JB, Dux PE. Improvements in Attention and Decision-Making Following Combined Behavioral Training and Brain Stimulation. Cereb Cortex. 2017;27(7):3675-82. | |
| 161 | Coffman BA, Trumbo MC, Flores RA, Garcia CM, van der Merwe AJ, Wassermann EM, et al. Impact of tDCS on performance and learning of target detection: interaction with stimulus characteristics and experimental design. Neuropsychologia. 2012;50(7):1594-602. | |
| 162 | Ellison A, Ball KL, Moseley P, Dowsett J, Smith DT, Weis S, et al. Functional interaction between right parietal and bilateral frontal cortices during visual search tasks revealed using functional magnetic imaging and transcranial direct current stimulation. PLoS One. 2014;9(4):e93767. | |
| 163 | Reinhart RM, Woodman GF. Enhancing long-term memory with stimulation tunes visual attention in one trial. Proc Natl Acad Sci U S A. 2015;112(2):625-30. | |
| 164 | Wagner J, Lo Monaco S, Conto F, Parrott D, Battelli L, Rusconi E. Effects of transcranial direct current stimulation over the posterior parietal cortex on novice X-ray screening performance. Cortex. 2020;132:1-14. | |
| 165 | Grasso PA, Tonolli E, Miniussi C. Effects of different transcranial direct current stimulation protocols on visuo-spatial contextual learning formation: evidence of homeostatic regulatory mechanisms. Sci Rep. 2020;10(1):4622. | |
| 166 | Bolognini N, Fregni F, Casati C, Olgiati E, Vallar G. Brain polarization of parietal cortex augments training-induced improvement of visual exploratory and attentional skills. Brain Res. 2010;1349:76-89. | |
| 167 | Ellison A, Ball KL, Lane AR. The Behavioral Effects of tDCS on Visual Search Performance Are Not Influenced by the Location of the Reference Electrode. Front Neurosci. 2017;11:520. | |
| **Working Memory** | | |
| **Ref no.** | | **Citation** |
| 57 | | Zivanovic M, Paunovic D, Konstantinovic U, Vulic K, Bjekic J, Filipovic SR. The effects of offline and online prefrontal vs parietal transcranial direct current stimulation (tDCS) on verbal and spatial working memory. Neurobiol Learn Mem. 2021;179:107398. |
| 168 | | Nikolin S, Loo CK, Bai S, Dokos S, Martin DM. Focalised stimulation using high definition transcranial direct current stimulation (HD-tDCS) to investigate declarative verbal learning and memory functioning. Neuroimage. 2015;117:11-9. |
| 169 | | Naka M, Matsuzawa D, Ishii D, Hamada H, Uchida T, Sugita K, et al. Differential effects of high-definition transcranial direct current stimulation on verbal working memory performance according to sensory modality. Neurosci Lett. 2018;687:131-6. |
| 170 | | Pupikova M, Simko P, Gajdos M, Rektorova I. Modulation of Working Memory and Resting-State fMRI by tDCS of the Right Frontoparietal Network. Neural Plast. 2021;2021:5594305. |
| 171 | | Murphy OW, Hoy KE, Wong D, Bailey NW, Fitzgerald PB, Segrave RA. Transcranial random noise stimulation is more effective than transcranial direct current stimulation for enhancing working memory in healthy individuals: Behavioural and electrophysiological evidence. Brain Stimul. 2020;13(5):1370-80. |
| 172 | | Karthikeyan R, Smoot MR, Mehta RK. Anodal tDCS augments and preserves working memory beyond time-on-task deficits. Sci Rep. 2021;11(1):19134. |
| 173 | | Caulfield KA, Indahlastari A, Nissim NR, Lopez JW, Fleischmann HH, Woods AJ, et al. Electric Field Strength From Prefrontal Transcranial Direct Current Stimulation Determines Degree of Working Memory Response: A Potential Application of Reverse-Calculation Modeling? Neuromodulation. 2022;25(4):578-87. |
| 174 | | Assecondi S, Hu R, Eskes G, Pan X, Zhou J, Shapiro K. Impact of tDCS on working memory training is enhanced by strategy instructions in individuals with low working memory capacity. Scientific Reports. 2021;11(1):5531. |
| 175 | | Ankri YLE, Braw Y, Luboshits G, Meiron O. The effects of stress and transcranial direct current stimulation (tDCS) on working memory: A randomized controlled trial. Cogn Affect Behav Neurosci. 2020;20(1):103-14. |
| 176 | | Abellaneda-Perez K, Vaque-Alcazar L, Perellon-Alfonso R, Bargallo N, Kuo MF, Pascual-Leone A, et al. Differential tDCS and tACS Effects on Working Memory-Related Neural Activity and Resting-State Connectivity. Front Neurosci. 2019;13:1440. |
| 177 | | Zwissler B, Sperber C, Aigeldinger S, Schindler S, Kissler J, Plewnia C. Shaping memory accuracy by left prefrontal transcranial direct current stimulation. J Neurosci. 2014;34(11):4022-6. |
| 178 | | Hussey EK, Fontes EB, Ward N, Westfall DR, Kao SC, Kramer AF, et al. Combined and Isolated Effects of Acute Exercise and Brain Stimulation on Executive Function in Healthy Young Adults. J Clin Med. 2020;9(5). |
| 179 | | Chrysikou EG, Gorey C, Aupperle RL. Anodal transcranial direct current stimulation over right dorsolateral prefrontal cortex alters decision making during approach-avoidance conflict. Soc Cogn Affect Neurosci. 2017;12(3):468-75. |
| 180 | | Boehringer A, Macher K, Dukart J, Villringer A, Pleger B. Cerebellar transcranial direct current stimulation modulates verbal working memory. Brain Stimul. 2013;6(4):649-53. |
| 181 | | Baumert A, Buchholz N, Zinkernagel A, Clarke P, MacLeod C, Osinsky R, et al. Causal underpinnings of working memory and Stroop interference control: Testing the effects of anodal and cathodal tDCS over the left DLPFC. Cogn Affect Behav Neurosci. 2020;20(1):34-48. |
| 182 | | Au J, Katz B, Buschkuehl M, Bunarjo K, Senger T, Zabel C, et al. Enhancing Working Memory Training with Transcranial Direct Current Stimulation. J Cogn Neurosci. 2016;28(9):1419-32. |
| **Vigilance** | |  |
| **Ref no.** | | **Citation** |
| 71 | | McIntire LK, McKinley RA, Goodyear C, Nelson J. A comparison of the effects of transcranial direct current stimulation and caffeine on vigilance and cognitive performance during extended wakefulness. Brain Stimul. 2014;7(4):499-507. |
| 168 | | Nikolin S, Loo CK, Bai S, Dokos S, Martin DM. Focalised stimulation using high definition transcranial direct current stimulation (HD-tDCS) to investigate declarative verbal learning and memory functioning. Neuroimage. 2015;117:11-9. |
| 169 | | Naka M, Matsuzawa D, Ishii D, Hamada H, Uchida T, Sugita K, et al. Differential effects of high-definition transcranial direct current stimulation on verbal working memory performance according to sensory modality. Neurosci Lett. 2018;687:131-6. |
| 183 | | van Schouwenburg MR, Sligte IG, Giffin MR, Günther F, Koster D, Spronkers FS, et al. Effects of Midfrontal Brain Stimulation on Sustained Attention. Journal of Cognitive Enhancement. 2021;5(1):62-72. |
| 184 | | Ironside M, O'Shea J, Cowen PJ, Harmer CJ. Frontal Cortex Stimulation Reduces Vigilance to Threat: Implications for the Treatment of Depression and Anxiety. Biol Psychiatry. 2016;79(10):823-30. |
| 185 | | Hanken K, Bosse M, Mohrke K, Eling P, Kastrup A, Antal A, et al. Counteracting Fatigue in Multiple Sclerosis with Right Parietal Anodal Transcranial Direct Current Stimulation. Front Neurol. 2016;7:154. |
| 186 | | Plewnia C, Zwissler B, Langst I, Maurer B, Giel K, Kruger R. Effects of transcranial direct current stimulation (tDCS) on executive functions: influence of COMT Val/Met polymorphism. Cortex. 2013;49(7):1801-7. |
| 187 | | McIntire LK, McKinley RA, Nelson JM, Goodyear C. Transcranial direct current stimulation versus caffeine as a fatigue countermeasure. Brain Stimul. 2017;10(6):1070-8. |
| 188 | | Manuel AL, David AW, Bikson M, Schnider A. Frontal tDCS modulates orbitofrontal reality filtering. Neuroscience. 2014;265:21-7. |
| 189 | | Luna FG, Roman-Caballero R, Barttfeld P, Lupianez J, Martin-Arevalo E. A High-Definition tDCS and EEG study on attention and vigilance: Brain stimulation mitigates the executive but not the arousal vigilance decrement. Neuropsychologia. 2020;142:107447. |
| 190 | | Jacoby N, Lavidor M. Null tDCS Effects in a Sustained Attention Task: The Modulating Role of Learning. Front Psychol. 2018;9:476. |
| 191 | | Filmer HL, Ehrhardt SE, Bollmann S, Mattingley JB, Dux PE. Accounting for individual differences in the response to tDCS with baseline levels of neurochemical excitability. Cortex. 2019;115:324-34. |
| 192 | | Coulborn S, Bowman H, Miall RC, Fernandez-Espejo D. Effect of tDCS Over the Right Inferior Parietal Lobule on Mind-Wandering Propensity. Front Hum Neurosci. 2020;14:230. |
| 193 | | Brunnauer A, Segmiller FM, Loschner S, Grun V, Padberg F, Palm U. The Effects of Transcranial Direct Current Stimulation (tDCS) on Psychomotor and Visual Perception Functions Related to Driving Skills. Front Behav Neurosci. 2018;12:16. |
| 194 | | Borragan G, Gilson M, Guerrero-Mosquera C, Di Ricci E, Slama H, Peigneux P. Transcranial Direct Current Stimulation Does Not Counteract Cognitive Fatigue, but Induces Sleepiness and an Inter-Hemispheric Shift in Brain Oxygenation. Front Psychol. 2018;9:2351. |
| **Inhibition** | |  |
| **Ref no.** | | **Citation** |
| 178 | | Hussey EK, Fontes EB, Ward N, Westfall DR, Kao SC, Kramer AF, et al. Combined and Isolated Effects of Acute Exercise and Brain Stimulation on Executive Function in Healthy Young Adults. J Clin Med. 2020;9(5). |
| 181 | | Baumert A, Buchholz N, Zinkernagel A, Clarke P, MacLeod C, Osinsky R, et al. Causal underpinnings of working memory and Stroop interference control: Testing the effects of anodal and cathodal tDCS over the left DLPFC. Cogn Affect Behav Neurosci. 2020;20(1):34-48. |
| 186 | | Plewnia C, Zwissler B, Langst I, Maurer B, Giel K, Kruger R. Effects of transcranial direct current stimulation (tDCS) on executive functions: influence of COMT Val/Met polymorphism. Cortex. 2013;49(7):1801-7. |
| 195 | | Angius L, Santarnecchi E, Pascual-Leone A, Marcora SM. Transcranial Direct Current Stimulation over the Left Dorsolateral Prefrontal Cortex Improves Inhibitory Control and Endurance Performance in Healthy Individuals. Neuroscience. 2019;419:34-45. |
| 196 | | Thomas F, Pixa NH, Berger A, Cheng MY, Doppelmayr M, Steinberg F. Neither Cathodal nor Anodal Transcranial Direct Current Stimulation on the Left Dorsolateral Prefrontal Cortex alone or Applied During Moderate Aerobic Exercise Modulates Executive Function. Neuroscience. 2020;443:71-83. |
| 197 | | Oldrati V, Patricelli J, Colombo B, Antonietti A. The role of dorsolateral prefrontal cortex in inhibition mechanism: A study on cognitive reflection test and similar tasks through neuromodulation. Neuropsychologia. 2016;91:499-508. |
| 198 | | Kuehne M, Schmidt K, Heinze HJ, Zaehle T. Modulation of Emotional Conflict Processing by High-Definition Transcranial Direct Current Stimulation (HD-TDCS). Front Behav Neurosci. 2019;13:224. |
| 199 | | Hammer A, Mohammadi B, Schmicker M, Saliger S, Munte TF. Errorless and errorful learning modulated by transcranial direct current stimulation. BMC Neurosci. 2011;12:72. |
| 200 | | Gomez-Ariza CJ, Martin MC, Morales J. Tempering Proactive Cognitive Control by Transcranial Direct Current Stimulation of the Right (but Not the Left) Lateral Prefrontal Cortex. Front Neurosci. 2017;11:282. |
| 201 | | Gladwin TE, den Uyl TE, Fregni FF, Wiers RW. Enhancement of selective attention by tDCS: interaction with interference in a Sternberg task. Neurosci Lett. 2012;512(1):33-7. |
| 202 | | Friedrich J, Beste C. Paradoxical, causal effects of sensory gain modulation on motor inhibitory control - a tDCS, EEG-source localization study. Sci Rep. 2018;8(1):17486. |
| 203 | | Edgcumbe DR, Thoma V, Rivolta D, Nitsche MA, Fu CHY. Anodal transcranial direct current stimulation over the right dorsolateral prefrontal cortex enhances reflective judgment and decision-making. Brain Stimul. 2019;12(3):652-8. |
| 204 | | Dubreuil-Vall L, Chau P, Ruffini G, Widge AS, Camprodon JA. tDCS to the left DLPFC modulates cognitive and physiological correlates of executive function in a state-dependent manner. Brain Stimul. 2019;12(6):1456-63. |
| 205 | | Denis G, Zory R, Radel R. Testing the role of cognitive inhibition in physical endurance using high-definition transcranial direct current stimulation over the prefrontal cortex. Hum Mov Sci. 2019;67:102507. |
| 206 | | Boudewyn M, Roberts BM, Mizrak E, Ranganath C, Carter CS. Prefrontal transcranial direct current stimulation (tDCS) enhances behavioral and EEG markers of proactive control. Cogn Neurosci. 2019;10(2):57-65. |

**Table S2.** Occurrence and central tendency of each stimulation/design parameter (given as number of effects for categorical factors and the median, min, and max for numeric variables).

| **Parameter Name** | **Motor**  **Performance** | **Visual**  **Search** | **Working**  **Memory** | **Vigilance** | **Inhibition** |
| --- | --- | --- | --- | --- | --- |
| **Target** | Cerebellum: 30  DLPFC: 33  M1: 160  Parietal: 14  PFC: 16 | DLPFC: 16  FEF: 14  IFC: 6  MFC: 6  MFG: 4  PFC: 16  PPC: 110  Visual: 4 | Cerebellum: 2  DLPFC: 90  MFG: 1  MTL: 2  PPC | DLPFC: 51  IPL: 10  MTL: 2  PFC: 2  Parietal: 2  PFC: 10  PPC: 12  Temporal: 2 | DLPFC: 75  IFC: 8  Parietal: 1 |
| **Laterality** | Bilateral: 33  Left: 151  Right: 69 | Bilateral: 14  Left: 88  Right: 74 | Left: 78  Right: 37 | Bilateral: 12  Left: 41  Right: 40 | Left: 60  Right: 24 |
| **Electrode count** | M = 2  Min = 2  Max = 9 | M = 2  Min = 2  Max = 3 | M = 2  Min = 2  Max = 5 | M =3  Min = 2  Max = 10 | M = 2  Min = 2  Max = 5 |
| **Individualization** | No: 208  Yes: 45 | NA | No: 110  Yes: 5 | NA | NA |
| **Intensity (mA)** | M = 1  Min = 0.25  Max = 2.1 | M = 1  Min = 0.61  Max = 3 | M = 1  Min = 1  Max = 2 | M = 1.5  Min = 0.7  Max = 2 | M = 1.75  Min = 1  Max = 5 |
| **Polarity** | Anodal: 191  Cathodal: 62 | Anodal: 91  Cathodal: 85 | Anodal: 103  Cathodal: 12 | Anodal: 83  Cathodal: 10 | Anodal: 56  Cathodal: 28 |
| **Duration (min)** | M = 20  Min = 10  Max = 30 | M = 15  Min = 10  Max = 30 | M = 15  Min = 5  Max = 30 | M = 20  Min = 9  Max = 30 | M = 20  Min = 10  Max = 30 |
| **Timing** | Mixed: 20  Offline: 138  Online: 95 | Mixed: 15  Offline: 93  Online: 68 | Mixed: 27  Offline: 41  Online: 47 | Mixed: 14  Offline: 31  Online: 48 | Mixed: 22  Offline: 44  Online: 18 |
| **Session count** | M = 1  Min = 1  Max = 20 | M = 1  Min = 1  Max = 4 | M = 1  Min = 1  Max = 10 | M = 1  Min = 1  Max = 3 | M = 1  Min = 1  Max = 1 |
| **Design** | Between: 130  Within: 123 | Between: 108  Within: 68 | Between: 64  Within: 51 | Between: 37  Within: 56 | Between: 42  Within: 42 |


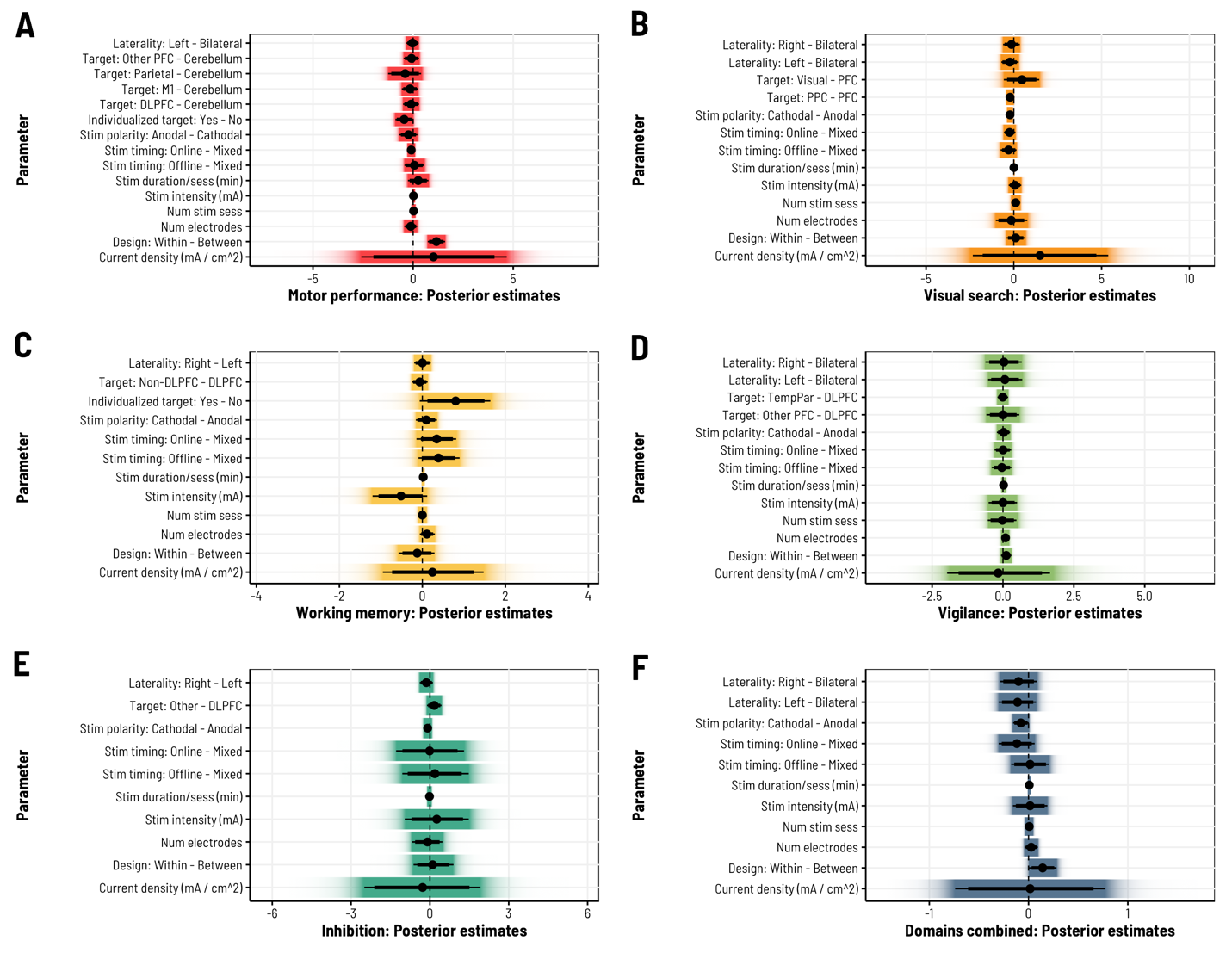


**Figure S1.** Contrasting estimates of multiple neurostimulation and experimental parameters on behavioral performance outcomes, both within-domain and between-domain. Here we include estimated current density over the target brain region (in mA / cm^2^) for each domain, where it was possible to calculate. Point intervals give 90-95% credibility intervals (i.e., highest density intervals) around the median posterior estimates (thick lines = 90% CI; thin lines = 95% CI; solid gradient areas indicate the 95% CI). Generally-speaking, current density did not show any relationship with behavioral outcomes and had the most posterior uncertainty, relative to other parameters. **(A)** Motor performance, **(B)** Visual search, **(C)** Working memory, **(D)** Vigilance, **(E)** Inhibition, **(F)** Domain-General.

**Table S3.** Summary of model comparison outcomes for two-way interaction models. For each exploratory model, *ELPD_Diff_* gives the mean difference in the expected log pointwise predictive density derived through Bayesian approximate leave-one-out cross validation. Positive values indicate a preference for the proposed interaction model (i.e., better predictive performance); negative values indicate a preference for the base model with only additive terms. *SE_Diff_* gives the standard error of the difference, and the ratio of the two can be used to approximate the significance of the difference (akin to a *Z*-statistic, where values > 2 indicate a nontrivial improvement in predictive performance when adding the proposed interaction term). No two-way interaction model met this criterion.

| **Candidate Model** | **Top Model** | ***ELPD_Diff_*** | ***SE_Diff_*** | ***ELPD_Diff_ / SE_Diff_*** |
| --- | --- | --- | --- | --- |
| Stim Polarity x Target Laterality | Base Model | -0.694 | 0.977 | -0.710 |
| Num Stim Sess x Target Laterality | Base Model | -2.387 | 1.767 | -1.351 |
| Stim Duration x Target Laterality | Interaction Model | 0.127 | 1.873 | 0.068 |
| Stim Intensity x Target Laterality | Base Model | -0.963 | 1.011 | -0.952 |
| Stim Timing x Target Laterality | Base Model | -0.720 | 2.750 | -0.262 |
| Num Electrodes x Target Laterality | Base Model | -0.428 | 2.128 | -0.201 |
| Design Type x Target Laterality | Interaction Model | 0.646 | 3.396 | 0.190 |
| Num Stim Sess x Stim Polarity | Interaction Model | 0.968 | 1.528 | 0.633 |
| Stim Duration x Stim Polarity | Base Model | -0.325 | 0.919 | -0.353 |
| Stim Intensity x Stim Polarity | Base Model | -0.882 | 0.344 | -2.562 |
| Stim Timing x Stim Polarity | Base Model | -1.582 | 0.920 | -1.719 |
| Num Electrodes x Stim Polarity | Base Model | -0.136 | 0.896 | -0.152 |
| Design Type x Stim Polarity | Interaction Model | 0.358 | 1.047 | 0.342 |
| Stim Duration x Num Stim Sess | Interaction Model | 0.905 | 3.161 | 0.286 |
| Stim Intensity x Num Stim Sess | Interaction Model | 0.290 | 0.932 | 0.311 |
| Stim Timing x Num Stim Sess | Base Model | -1.661 | 2.073 | -0.801 |
| Num Electrodes x Num Stim Sess | Base Model | -0.057 | 0.634 | -0.090 |
| Design Type x Num Stim Sess | Base Model | -0.148 | 0.993 | -0.149 |
| Stim Intensity x Stim Duration | Base Model | -0.287 | 0.483 | -0.595 |
| Stim Timing x Stim Duration | Base Model | -0.370 | 0.859 | -0.431 |
| Num Electrodes x Stim Duration | Interaction Model | 0.117 | 0.700 | 0.167 |
| Design Type x Stim Duration | Interaction Model | 0.183 | 0.396 | 0.463 |
| Stim Timing x Stim Intensity | Base Model | -0.238 | 1.311 | -0.182 |
| Num Electrodes x Stim Intensity | Interaction Model | 0.046 | 0.232 | 0.199 |
| Design Type x Stim Intensity | Interaction Model | 4.930 | 2.821 | 1.748 |
| Num Electrodes x Stim Timing | Interaction Model | 5.844 | 4.449 | 1.313 |
| Design Type x Stim Timing | Interaction Model | 1.005 | 1.625 | 0.618 |
| Design Type x Num Electrodes | Interaction Model | 1.880 | 2.074 | 0.906 |

**Table S4.** Summary of model comparison outcomes for three-way interaction models. For each exploratory model, *ELPD_Diff_* gives the mean difference in the expected log pointwise predictive density derived through Bayesian approximate leave-one-out cross validation. Positive values indicate a preference for the proposed interaction model (i.e., better predictive performance); negative values indicate a preference for the base model with only additive terms. *SE_Diff_* gives the standard error of the difference, and the ratio of the two can be used to approximate the significance of the difference (akin to a *Z*-statistic, where values > 2 indicate a nontrivial improvement in predictive performance when adding the proposed interaction term). Several three-way interaction models met this criterion, bolded and italicized below.

| **Candidate Model** | **Top Model** | ***ELPD_Diff_*** | ***SE_Diff_*** | ***ELPD_Diff_ / SE_Diff_*** |
| --- | --- | --- | --- | --- |
| Num Stim Sess x Stim Polarity x Target Laterality | Base Model | -4.571 | 3.309 | -1.381 |
| Stim Duration x Stim Polarity x Target Laterality | Base Model | -4.467 | 2.638 | -1.693 |
| Stim Intensity x Stim Polarity x Target Laterality | Base Model | -7.466 | 1.242 | -6.010 |
| Stim Timing x Stim Polarity x Target Laterality | Base Model | -11.922 | 3.865 | -3.085 |
| Num Electrodes x Stim Polarity x Target Laterality | Base Model | -23.571 | 5.509 | -4.279 |
| Design Type x Stim Polarity x Target Laterality | Interaction Model | 0.266 | 4.516 | 0.059 |
| Num Stim Sess x Stim Duration x Target Laterality | Base Model | -6.457 | 3.970 | -1.626 |
| Num Stim Sess x Stim Intensity x Target Laterality | Base Model | -5.816 | 2.655 | -2.190 |
| Num Stim Sess x Stim Timing x Target Laterality | Base Model | -200.353 | 17.132 | -11.694 |
| Design Type x Num Stim Sess x Target Laterality | Base Model | -2.469 | 5.892 | -0.419 |
| Stim Intensity x Stim Duration x Target Laterality | Base Model | -176.908 | 11.665 | -15.166 |
| Stim Duration x Stim Timing x Target Laterality | Base Model | -150.205 | 11.004 | -13.650 |
| Num Electrodes x Stim Duration x Target Laterality | Base Model | -3.442 | 3.453 | -0.997 |
| Design Type x Stim Duration x Target Laterality | Interaction Model | 1.157 | 6.778 | 0.171 |
| Num Stim Sess x Stim Duration x Stim Polarity | Interaction Model | 2.945 | 3.102 | 0.949 |
| Num Stim Sess x Stim Intensity x Stim Polarity | Base Model | -0.436 | 2.061 | -0.211 |
| Num Stim Sess x Stim Timing x Stim Polarity | Base Model | -5.463 | 2.610 | -2.093 |
| Num Electrodes x Num Stim Sess x Stim Polarity | Base Model | -31.651 | 5.982 | -5.291 |
| Design Type x Num Stim Sess x Stim Polarity | Interaction Model | 2.354 | 2.943 | 0.800 |
| Stim Intensity x Stim Duration x Stim Polarity | Base Model | -3.496 | 1.130 | -3.093 |
| Stim Duration x Stim Timing x Stim Polarity | Base Model | -3.041 | 2.814 | -1.081 |
| Design Type x Stim Duration x Stim Polarity | Interaction Model | 0.859 | 2.143 | 0.401 |
| Stim Intensity x Stim Timing x Stim Polarity | Base Model | -6.102 | 2.116 | -2.884 |
| Num Electrodes x Stim Intensity x Stim Polarity | Base Model | -1.830 | 1.729 | -1.059 |
| Design Type x Stim Intensity x Stim Polarity | Interaction Model | 3.941 | 2.964 | 1.330 |
| Num Electrodes x Stim Timing x Stim Polarity | Base Model | -31.402 | 7.316 | -4.292 |
| Design Type x Stim Timing x Stim Polarity | Base Model | -1.868 | 2.570 | -0.727 |
| Design Type x Num Electrodes x Stim Polarity | Base Model | -7.386E+18 | 7.386E+18 | -1.000 |
| Num Stim Sess x Stim Intensity x Stim Duration | Base Model | -2.939 | 4.150 | -0.708 |
| Num Stim Sess x Stim Duration x Stim Timing | Base Model | -4.119 | 5.562 | -0.741 |
| Num Electrodes x Num Stim Sess x Stim Duration | Base Model | -0.501 | 3.193 | -0.157 |
| Design Type x Num Stim Sess x Stim Duration | Base Model | -1.412 | 3.299 | -0.428 |
| Num Stim Sess x Stim Intensity x Stim Timing | Base Model | -1.017 | 3.134 | -0.324 |
| Num Electrodes x Num Stim Sess x Stim Intensity | Interaction Model | 0.022 | 0.909 | 0.024 |
| Design Type x Num Stim Sess x Stim Intensity | Interaction Model | 5.329 | 3.683 | 1.447 |
| Num Electrodes x Num Stim Sess x Stim Timing | Base Model | -31.620 | 17.935 | -1.763 |
| Design Type x Num Stim Sess x Stim Timing | Base Model | -105.643 | 14.833 | -7.122 |
| Num Electrodes x Num Stim Sess x Target Laterality | Base Model | -93.958 | 10.411 | -9.025 |
| Design Type x Num Electrodes x Num Stim Sess | Interaction Model | 0.424 | 3.069 | 0.138 |
| Stim Intensity x Stim Duration x Stim Timing | Base Model | -1.888 | 2.311 | -0.817 |
| Num Electrodes x Stim Intensity x Stim Duration | Interaction Model | 0.147 | 1.058 | 0.139 |
| ***Design Type x Stim Intensity x Stim Duration*** | ***Interaction Model*** | ***16.448*** | ***7.352*** | ***2.237*** |
| Num Electrodes x Stim Duration x Stim Timing | Interaction Model | 3.564 | 4.495 | 0.793 |
| Design Type x Stim Duration x Stim Timing | Base Model | -0.735 | 2.137 | -0.344 |
| ***Design Type x Num Electrodes x Stim Duration*** | ***Interaction Model*** | ***15.818*** | ***7.363*** | ***2.148*** |
| Num Electrodes x Stim Intensity x Target Laterality | Base Model | -5.671 | 2.856 | -1.986 |
| Design Type x Stim Intensity x Target Laterality | Interaction Model | 4.538 | 5.985 | 0.758 |
| Stim Intensity x Stim Timing x Target Laterality | Base Model | -172.785 | 11.626 | -14.862 |
| Num Electrodes x Stim Intensity x Stim Timing | Interaction Model | 4.357 | 7.482 | 0.582 |
| Design Type x Stim Intensity x Stim Timing | Interaction Model | 6.276 | 5.111 | 1.228 |
| ***Design Type x Num Electrodes x Stim Intensity*** | ***Interaction Model*** | ***23.287*** | ***11.403*** | ***2.042*** |
| Num Electrodes x Stim Timing x Target Laterality | Base Model | -165.216 | 11.660 | -14.169 |
| Design Type x Stim Timing x Target Laterality | Base Model | -132.073 | 14.904 | -8.861 |
| Design Type x Num Electrodes x Stim Timing | Interaction Model | 13.369 | 8.243 | 1.622 |
| Design Type x Num Electrodes x Target Laterality | Base Model | -1.369 | 4.901 | -0.279 |
| Num Electrodes x Stim Duration x Stim Polarity | Base Model | -319.103 | 19.998 | -15.957 |

**Table S5.** Differences in candidate model predictive performance for the Motor domain, derived via Bayesian approximate leave-one-out cross-validation. Differences in fit are presented with respect to the top listed model. *ELPD_Diff_* gives the mean difference in the expected log pointwise predictive densities (negative values thus indicate decrements in predictive performance relative to the top model); *SE_Diff_* gives the standard error of the difference. The top model significantly differs in performance from any other model where |*ELPD_Diff_* / *SE_Diff_*| > 2.

| **Candidate Model** | ***ELPD_Diff_*** | ***SE_Diff_*** | ***ELPD_Diff_ / SE_Diff_*** |
| --- | --- | --- | --- |
| Design Type x Num Electrodes x Stim Duration | — | — | — |
| Design Type x Num Electrodes x Stim Intensity | -1.678 | 2.126 | -0.789 |
| Design Type x Stim Intensity x Stim Duration | -5.079 | 3.190 | -1.592 |
| Base Model (Only Additive Terms) | -13.796 | 7.331 | -1.882 |

**Table S6.** Model summary for top motion performance candidate.

| **Fixed Predictors** | **Post. Med. Est.** | **Post. *SD*** | **95CI (*p*_d_)** |
| --- | --- | --- | --- |
| Intercept | -13.94 | 5.96 | -25.69 – -1.99 (.988) |
| Polarity: Anodal - Cathodal | -0.07 | 0.08 | -0.22 – 0.09 (.805) |
| Target: DLPFC - Cerebellum | -0.35 | 0.20 | -0.75 – 0.06 (.954) |
| Target: M1 - Cerebellum | -0.03 | 0.18 | -0.39 – 0.33 (.563) |
| Target: Parietal - Cerebellum | -0.02 | 0.20 | -0.42 – 0.37 (.546) |
| Target: Other PFC - Cerebellum | -0.54 | 0.38 | -1.31 – 0.22 (.920) |
| Individualized target: Yes - No | -0.22 | 0.19 | -0.61 – 0.16 (.871) |
| Laterality: Left - Bilateral | -0.12 | 0.18 | -0.47 – 0.24 (.748) |
| Laterality: Right - Bilateral | -0.11 | 0.15 | -0.40 – 0.18 (.765) |
| Number of stimulation sessions | -0.03 | 0.03 | -0.09 – 0.04 (.801) |
| Stimulation duration/session (min) | 0.70 | 0.28 | 0.13 – 1.26 (.991) |
| Stimulation intensity (mA) | -0.18 | 0.19 | -0.56 – 0.21 (.820) |
| Stimulation timing: Offline - Mixed | 0.24 | 0.23 | -0.22 – 0.68 (.850) |
| Stimulation timing: Online - Mixed | 0.12 | 0.22 | -0.32 – 0.55 (.699) |
| Number of electrodes in montage | 7.10 | 2.97 | 1.14 – 12.99 (.984) |
| Design: Within - Between | 30.37 | 11.57 | 7.22 – 53.53 (.994) |
| Design: Within - Between x  Number of electrodes in montage | -15.38 | 5.77 | -26.90 – -3.80 (.995) |
| Design: Within - Between x  Stimulation duration/session | -1.51 | 0.55 | -2.62 – -0.41 (.996) |
| Number of electrodes in montage x  Stimulation duration/session | -0.35 | 0.14 | -0.63 – -0.06 (.991) |
| Design: Within - Between x  Number of electrodes in montage x  Stimulation duration/session | 0.76 | 0.27 | 0.21 – 1.31 (.996) |
| **Random Effects (*N* studies = 42)** | | | |
| σ_StudyID_ (Intercept) | 0.56 | 0.08 | 0.42 – 0.74 |
| *N* total effects = 253 | | | |
| Marginal *R*^2^ = 0.23 [0.16, 0.32] | | | |
| Conditional *R*^2^ = 0.44 [0.38, 0.50] | | | |

**Table S7.** Differences in candidate model predictive performance for the Visual Search domain, derived via Bayesian approximate leave-one-out cross-validation. Differences in fit are presented with respect to the top listed model. *ELPD_Diff_* gives the mean difference in the expected log pointwise predictive densities (negative values thus indicate decrements in predictive performance relative to the top model); *SE_Diff_* gives the standard error of the difference. The top model significantly differs in performance from any other model where |*ELPD_Diff_* / *SE_Diff_*| > 2.

| **Candidate Model** | ***ELPD_Diff_*** | ***SE_Diff_*** | ***ELPD_Diff_ / SE_Diff_*** |
| --- | --- | --- | --- |
| Base Model (Only Additive Terms) | — | — | — |
| Design Type x Stim Intensity x Stim Duration | -0.099 | 1.589 | -0.062 |
| Design Type x Num Electrodes x Stim Duration | -624.797 | 6.581 | -94.946 |
| Design Type x Num Electrodes x Stim Intensity | -654.956 | 9.758 | -67.119 |

| **Fixed Predictors** | **Post. Med. Est.** | **Post. *SD*** | **95CI (*p*_d_)** |
| --- | --- | --- | --- |
| Intercept | 1.36 | 0.89 | -0.74 – 3.34 (.916) |
| Polarity: Anodal - Cathodal | -0.23 | 0.08 | -0.39 – -0.07 (.998) |
| Target: PPC - PFC | -0.18 | 0.10 | -0.38 – 0.01 (.968) |
| Target: Visual - PFC | 0.37 | 0.39 | -0.45 – 1.25 (.830) |
| Laterality: Left - Bilateral | -0.35 | 0.27 | -0.87 – 0.19 (.899) |
| Laterality: Right - Bilateral | -0.25 | 0.26 | -0.75 – 0.27 (.828) |
| Number of stimulation sessions | 0.07 | 0.10 | -0.15 – 0.29 (.751) |
| Stimulation duration/session (min) | -0.07 | 0.04 | -0.17 – 0.03 (.936) |
| Stimulation intensity (mA) | -0.91 | 0.47 | -1.90 – 0.10 (.963) |
| Stimulation timing: Offline - Mixed | -0.33 | 0.20 | -0.75 – 0.05 (.955) |
| Stimulation timing: Online - Mixed | -0.19 | 0.16 | -0.52 – 0.12 (.878) |
| Number of electrodes in montage | 0.03 | 0.35 | -0.76 – 0.83 (.536) |
| Design: Within - Between | -0.20 | 1.24 | -2.91 – 2.64 (.567) |
| Design: Within - Between x  Stimulation intensity | 0.36 | 0.90 | -1.63 – 2.29 (.655) |
| Design: Within - Between x  Stimulation duration/session | 0.04 | 0.09 | -0.15 – 0.23 (.693) |
| Stim intensity x  Stimulation duration/session | 0.05 | 0.03 | -0.003 – 0.11 (.969) |
| Design: Within - Between x  Stimulation intensity x  Stimulation duration/session | -0.04 | 0.05 | -0.15 – 0.08 (.762) |
| **Random Effects (*N* studies = 18)** | | | |
| σ_StudyID_ (Intercept) | 0.25 | 0.11 | 0.06 – 0.51 |
| *N* total effects = 176 | | | |
| Marginal *R*^2^ = 0.47 [0.33, 0.58] | | | |
| Conditional *R*^2^ = 0.51 [0.41, 0.60] | | | |

**Table S8.** Model summary for top visual search candidate.

**Table S9.** Differences in candidate model predictive performance for the Working Memory domain, derived via Bayesian approximate leave-one-out cross-validation. Differences in fit are presented with respect to the top listed model. *ELPD_Diff_* gives the mean difference in the expected log pointwise predictive densities (negative values thus indicate decrements in predictive performance relative to the top model); *SE_Diff_* gives the standard error of the difference. The top model significantly differs in performance from any other model where |*ELPD_Diff_* / *SE_Diff_*| > 2.

| **Candidate Model** | ***ELPD_Diff_*** | ***SE_Diff_*** | ***ELPD_Diff_ / SE_Diff_*** |
| --- | --- | --- | --- |
| Design Type x Stim Intensity x Stim Duration | — | — | — |
| Base Model (Only Additive Terms) | -3.101 | 2.499 | -1.241 |
| Design Type x Num Electrodes x Stim Intensity | -102.34 | 12.966 | -7.893 |
| Design Type x Num Electrodes x Stim Duration | -473.343 | 25.129 | -18.837 |

**Table S10.** Model summary for top working memory candidate.

| **Fixed Predictors** | **Post. Med. Est.** | **Post. *SD*** | **95CI (*p*_d_)** |
| --- | --- | --- | --- |
| Intercept | -2.85 | 1.22 | -5.43 – 0.04 (.973) |
| Polarity: Anodal - Cathodal | 0.19 | 0.12 | -0.04 – 0.43 (.942) |
| Target: Non-DLPFC - DLPFC | -0.03 | 0.10 | -0.22 – 0.16 (.620) |
| Individualized target: Yes - No | 0.69 | 0.29 | 0.09 – 1.29 (.986) |
| Laterality: Right - Left | 0.02 | 0.09 | -0.16 – 0.21 (.597) |
| Number of stimulation sessions | 0.04 | 0.03 | -0.03 – 0.11 (.889) |
| Stimulation duration/session (min) | 0.27 | 0.08 | 0.08 – 0.44 (.992) |
| Stimulation intensity (mA) | 1.78 | 0.77 | -0.05 – 3.38 (.973) |
| Stimulation timing: Offline - Mixed | 0.08 | 0.20 | -0.34 – 0.53 (.647) |
| Stimulation timing: Online - Mixed | 0.02 | 0.20 | -0.42 – 0.46 (.544) |
| Number of electrodes in montage | 0.14 | 0.06 | 0.02 – 0.27 (.989) |
| Design: Within - Between | 0.37 | 2.24 | -4.76 – 6.13 (.566) |
| Design: Within - Between x  Stimulation intensity | -0.42 | 1.42 | -4.10 – 2.87 (.620) |
| Design: Within - Between x  Stimulation duration/session | 0.18 | 0.15 | -0.20 – 0.50 (.858) |
| Stimulation intensity x  Stimulation duration/session | -0.15 | 0.05 | -0.25 – -0.04 (.991) |
| Design: Within - Between x  Stimulation intensity x  Stimulation duration/session | -0.09 | 0.09 | -0.28 – 0.13 (.829) |
| **Random Effects (*N* studies = 16)** | | | |
| σ_StudyID_ (Intercept) | 0.09 | 0.08 | 0.00 – 0.38 |
| *N* total effects = 115 | | | |
| Marginal *R*^2^ = 0.16 [0.08, 0.30] | | | |
| Conditional *R*^2^ = 0.15 [0.09, 0.22] | | | |

**Table S11.** Differences in candidate model predictive performance for the Vigilance domain, derived via Bayesian approximate leave-one-out cross-validation. Differences in fit are presented with respect to the top listed model. *ELPD_Diff_* gives the mean difference in the expected log pointwise predictive densities (negative values thus indicate decrements in predictive performance relative to the top model); *SE_Diff_* gives the standard error of the difference. The top model significantly differs in performance from any other model where |*ELPD_Diff_* / *SE_Diff_*| > 2.

| **Candidate Model** | ***ELPD_Diff_*** | ***SE_Diff_*** | ***ELPD_Diff_ / SE_Diff_*** |
| --- | --- | --- | --- |
| Base Model (Only Additive Terms) | — | — | — |
| Design Type x Stim Intensity x Stim Duration | -0.666 | 1.480 | -0.450 |
| Design Type x Num Electrodes x Stim Intensity | -0.671 | 2.081 | -0.322 |
| Design Type x Num Electrodes x Stim Duration | -0.760 | 2.148 | -0.354 |

**Table S12.** Model summary for top vigilance candidate.

| **Fixed Predictors** | **Post. Med. Est.** | **Post. *SD*** | **95CI (*p*_d_)** |
| --- | --- | --- | --- |
| Intercept | -1.70 | 10.12 | -24.40 – 19.40 (.568) |
| Polarity: Anodal - Cathodal | 0.04 | 0.12 | -0.20 – 0.28 (.634) |
| Target: Other PFC - DLPFC | 0.19 | 0.31 | -0.50 – 0.89 (.732) |
| Target: Temporal-Parietal - DLPFC | -0.01 | 0.10 | -0.20 – 0.18 (.528) |
| Laterality: Left - Bilateral | 0.95 | 0.98 | -1.01 – 3.05 (.837) |
| Laterality: Right - Bilateral | 0.81 | 0.83 | -0.93 – 2.62 (.834) |
| Number of stimulation sessions | 0.86 | 0.89 | -0.97 – 2.82 (.832) |
| Stimulation duration/session (min) | 0.06 | 0.49 | -0.96 – 1.16 (.547) |
| Stimulation intensity (mA) | 0.51 | 5.26 | -10.46 – 12.16 (.541) |
| Stimulation timing: Offline - Mixed | -0.03 | 0.23 | -0.50 – 0.43 (.546) |
| Stimulation timing: Online - Mixed | -0.02 | 0.16 | -0.36 – 0.31 (.557) |
| Number of electrodes in montage | 0.04 | 0.08 | -0.16 – 0.24 (.693) |
| Design: Within - Between | -14.06 | 11.56 | -41.34 – 13.84 (.873) |
| Design: Within - Between x  Stimulation intensity | 9.32 | 6.84 | -7.41 – 25.84 (.897) |
| Design: Within - Between x  Stimulation duration/session | 0.66 | 0.59 | -0.76 – 2.02 (.855) |
| Stimulation intensity x  Stimulation duration/session | -0.05 | 0.29 | -0.68 – 0.55 (.567) |
| Design: Within - Between x  Stimulation intensity x  Stimulation duration/session | -0.43 | 0.33 | -1.23 – 0.38 (.886) |
| **Random Effects (*N* studies = 15)** | | | |
| σ_StudyID_ (Intercept) | 0.17 | 0.15 | 0.01 – 0.55 |
| *N* total effeccts = 93 | | | |
| Marginal *R*^2^ = 0.43 [0.27, 0.55] | | | |
| Conditional *R*^2^ = 0.42 [0.28, 0.53] | | | |

**Table S13.** Differences in candidate model predictive performance for the Inhibition domain, derived via Bayesian approximate leave-one-out cross-validation. Differences in fit are presented with respect to the top listed model. *ELPD_Diff_* gives the mean difference in the expected log pointwise predictive densities (negative values thus indicate decrements in predictive performance relative to the top model); *SE_Diff_* gives the standard error of the difference. The top model significantly differs in performance from any other model where |*ELPD_Diff_* / *SE_Diff_*| > 2.

| **Candidate Model** | ***ELPD_Diff_*** | ***SE_Diff_*** | ***ELPD_Diff_ / SE_Diff_*** |
| --- | --- | --- | --- |
| Base Model (Only Additive Terms) | — | — | — |
| Design Type x Stim Intensity x Stim Duration | -81.191 | 12.117 | -6.701 |
| Design Type x Num Electrodes x Stim Duration | -156.164 | 15.967 | -9.780 |
| Design Type x Num Electrodes x Stim Intensity | -174.519 | 15.943 | -10.946 |

**Table S14.** Differences in candidate model predictive performance for the combined domains, derived via Bayesian approximate leave-one-out cross-validation. Differences in fit are presented with respect to the top listed model. *ELPD_Diff_* gives the mean difference in the expected log pointwise predictive densities (negative values thus indicate decrements in predictive performance relative to the top model); *SE_Diff_* gives the standard error of the difference. The top model significantly differs in performance from any other model where |*ELPD_Diff_* / *SE_Diff_*| > 2.

| **Candidate Model** | ***ELPD_Diff_*** | ***SE_Diff_*** | ***ELPD_Diff_ / SE_Diff_*** |
| --- | --- | --- | --- |
| Design Type x Num Electrodes x Stim Intensity | — | — | — |
| Design Type x Stim Intensity x Stim Duration | -6.904 | 4.839 | -1.427 |
| Design Type x Num Electrodes x Stim Duration | -7.038 | 4.703 | -1.497 |
| Base Model (Only Additive Terms) | -23.029 | 11.329 | -2.033 |


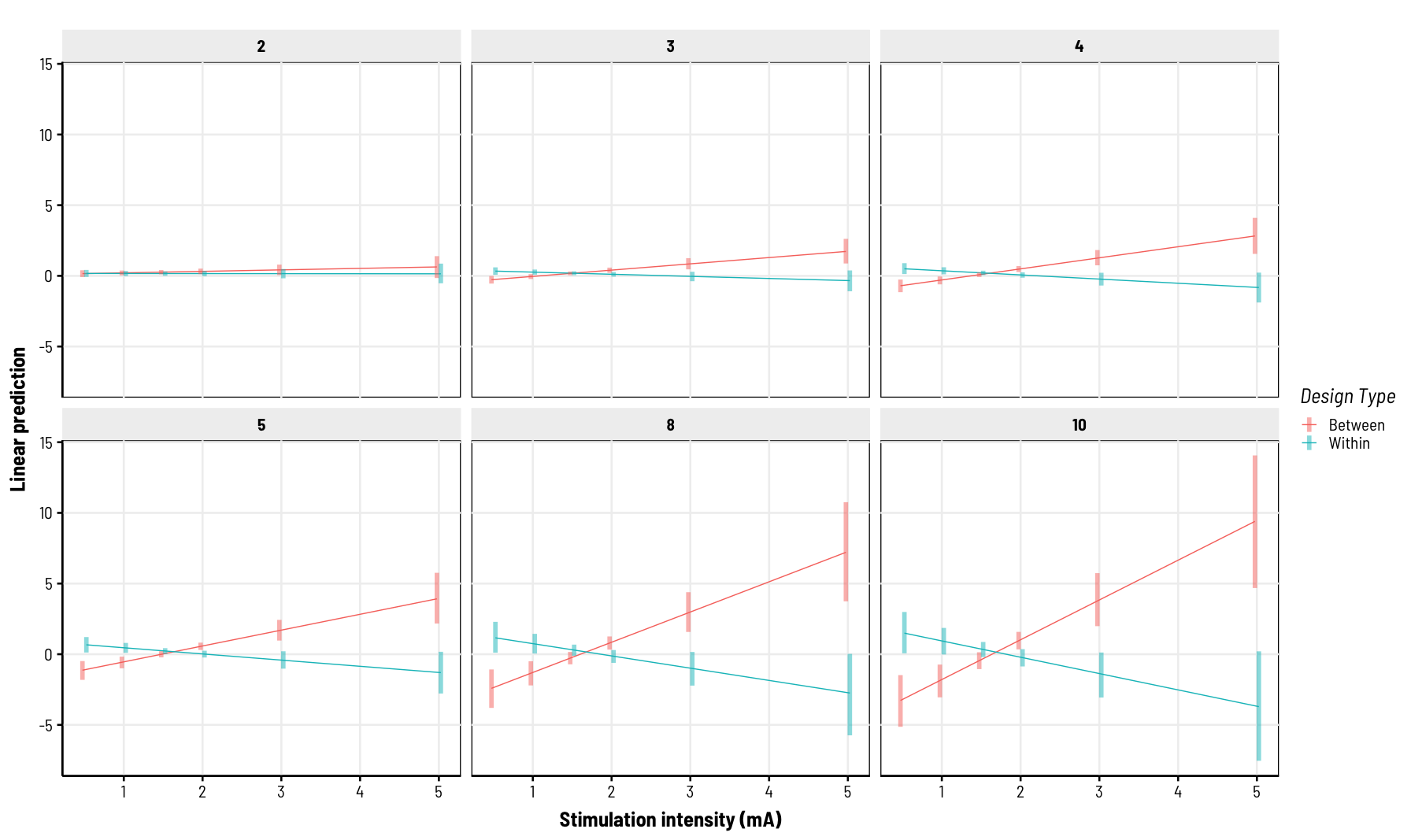


**Figure S2.** Posterior predictions for a three-way interaction between Design Type x Num Electrodes x Stim Intensity (all domains combined). For visualization, stimulation intensities (along the *x*-axis) were sampled at 0.50, 1, 1.5, 2, 3, and 5 mA; montage sizes (individual facets/tiles) were sampled at 2, 3, 4, 5, 8, and 10 electrodes. Error bars give 95% highest posterior density intervals. These trends suggest that larger montages coupled with stronger stimulation intensities may produce larger behavioral effects in between-subject designs, but this is reversed under within-subject designs.

**Table S15.** Model summary for top candidate model with domains combined.

| **Fixed Predictors** | **Post. Med. Est.** | **Post. *SD*** | **95CI (*p*_d_)** |
| --- | --- | --- | --- |
| Intercept | 0.45 | 0.33 | -0.20 – 1.10 (.914) |
| Polarity: Anodal - Cathodal | -0.07 | 0.04 | -0.15 – 0.01 (.956) |
| Laterality: Left - Bilateral | -0.14 | 0.10 | -0.33 – 0.05 (.920) |
| Laterality: Right - Bilateral | -0.12 | 0.09 | -0.30 – 0.07 (.887) |
| Number of stimulation sessions | 0.00 | 0.02 | -0.05 – 0.04 (.590) |
| Stimulation duration/session (min) | 0.00 | 0.01 | -0.01 – 0.02 (.677) |
| Stimulation intensity (mA) | -0.14 | 0.17 | -0.48 – 0.19 (.808) |
| Stimulation timing: Offline - Mixed | 0.00 | 0.10 | -0.20 – 0.19 (.519) |
| Stimulation timing: Online - Mixed | -0.12 | 0.09 | -0.31 – 0.06 (.906) |
| Number of electrodes in montage | -0.18 | 0.12 | -0.43 – 0.07 (.923) |
| Design: Within - Between | -1.60 | 0.47 | -2.57 – -0.69 (1.00) |
| Design: Within - Between x  Number of electrodes in montage | 0.84 | 0.16 | 0.53 – 1.16 (1.00) |
| Design: Within - Between x  Stimulation intensity | 0.86 | 0.28 | 0.32 – 1.43 (.999) |
| Number of electrodes in montage x  Stimulation intensity | 0.10 | 0.07 | -0.04 – 0.23 (.923) |
| Design: Within - Between x  Number of electrodes in montage x  Stimulation intensity | -0.48 | 0.10 | -0.69 – -0.29 (1.00) |
| **Random Effects (*N* studies = 106)** | | | |
| σ_StudyID_ (Intercept) | 0.43 | 0.04 | 0.36 – 0.52 |
| *N* total effects = 721 | | | |
| Marginal *R*^2^ = 0.02 [0.07, 0.17] | | | |
| Conditional *R*^2^ = 0.33 [0.29, 0.37] | | | |


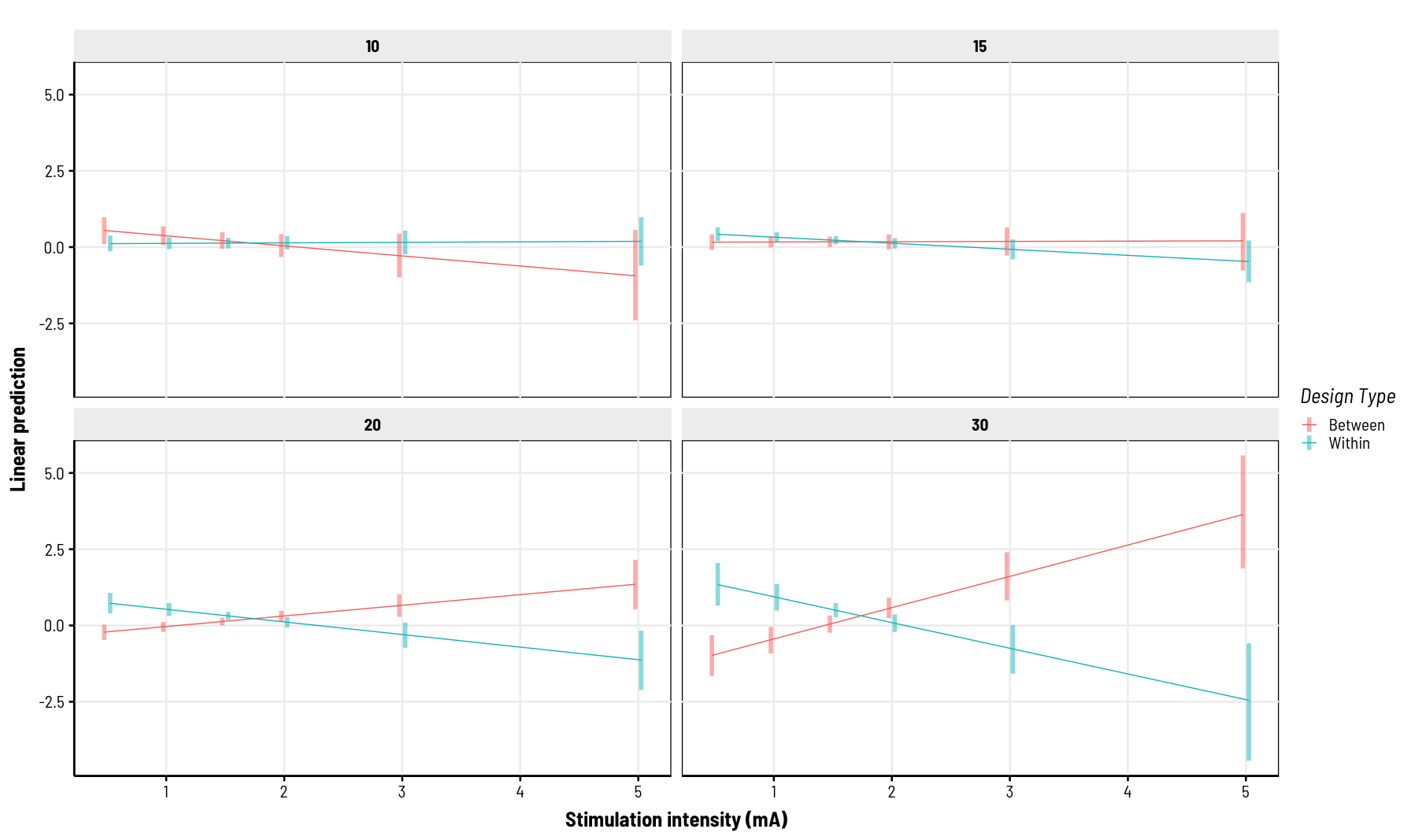


**Figure S3.** Posterior predictions for a three-way interaction between Design Type x Stim Intensity x Stim Duration (all domains combined). For visualization, stimulation intensities (along the *x*-axis) were sampled at 0.50, 1, 1.5, 2, 3, and 5 mA; stimulation durations (individual facets/tiles) were sampled at 10, 15, 20, and 30 minutes. Error bars give 95% highest posterior density intervals. These trends suggest that longer and stronger stimulation sessions may produce larger behavioral effects in between-subject designs, but this is reversed under within-subject designs.

| **Fixed Predictors** | **Post. Med. Est.** | **Post. *SD*** | **95CI (*p*_d_)** |
| --- | --- | --- | --- |
| Intercept | 0.54 | 0.41 | -0.25 – 1.35 (.911) |
| Polarity: Anodal - Cathodal | -0.08 | 0.04 | -0.15 – 0.00 (.973) |
| Laterality: Left - Bilateral | -0.15 | 0.09 | -0.33 – 0.04 (.941) |
| Laterality: Right - Bilateral | -0.12 | 0.09 | -0.30 – 0.06 (.902) |
| Number of stimulation sessions | -0.00 | 0.02 | -0.04 – 0.04 (.505) |
| Stimulation duration/session (min) | -0.01 | 0.02 | -0.06 – 0.03 (.718) |
| Stimulation intensity (mA) | -0.28 | 0.24 | -0.76 – 0.19 (.880) |
| Stimulation timing: Offline - Mixed | -0.01 | 0.10 | -0.21 – 0.18 (.557) |
| Stimulation timing: Online - Mixed | -0.12 | 0.09 | -0.31 – 0.06 (.910) |
| Number of electrodes in montage | 0.00 | 0.03 | -0.05 – 0.06 (.517) |
| Design: Within - Between | -2.54 | 0.74 | -4.07 – -1.11 (1.00) |
| Design: Within - Between x  Stimulation intensity | 1.46 | 0.46 | 0.59 – 2.39 (.999) |
| Design: Within - Between x  Stimulation duration/session | 0.19 | 0.04 | 0.11 – 0.28 (1.00) |
| Stimulation intensity x  Stimulation duration/session | 0.01 | 0.01 | -0.02 – 0.04 (.811) |
| Design: Within - Between x  Stimulation intensity x  Stimulation duration/session | -0.11 | 0.03 | -0.17 – -0.06 (1.00) |
| **Random Effects (*N* studies = 106)** | | | |
| σ_StudyID_ (Intercept) | 0.42 | 0.04 | 0.34 – 0.51 |
| *N* total effects = 721 | | | |
| Marginal *R*^2^ = 0.13 [0.08, 0.20] | | | |
| Conditional *R*^2^ = 0.32 [0.27, 0.36] | | | |

**Table S16.** Model summary for second-best candidate model with domains combined.


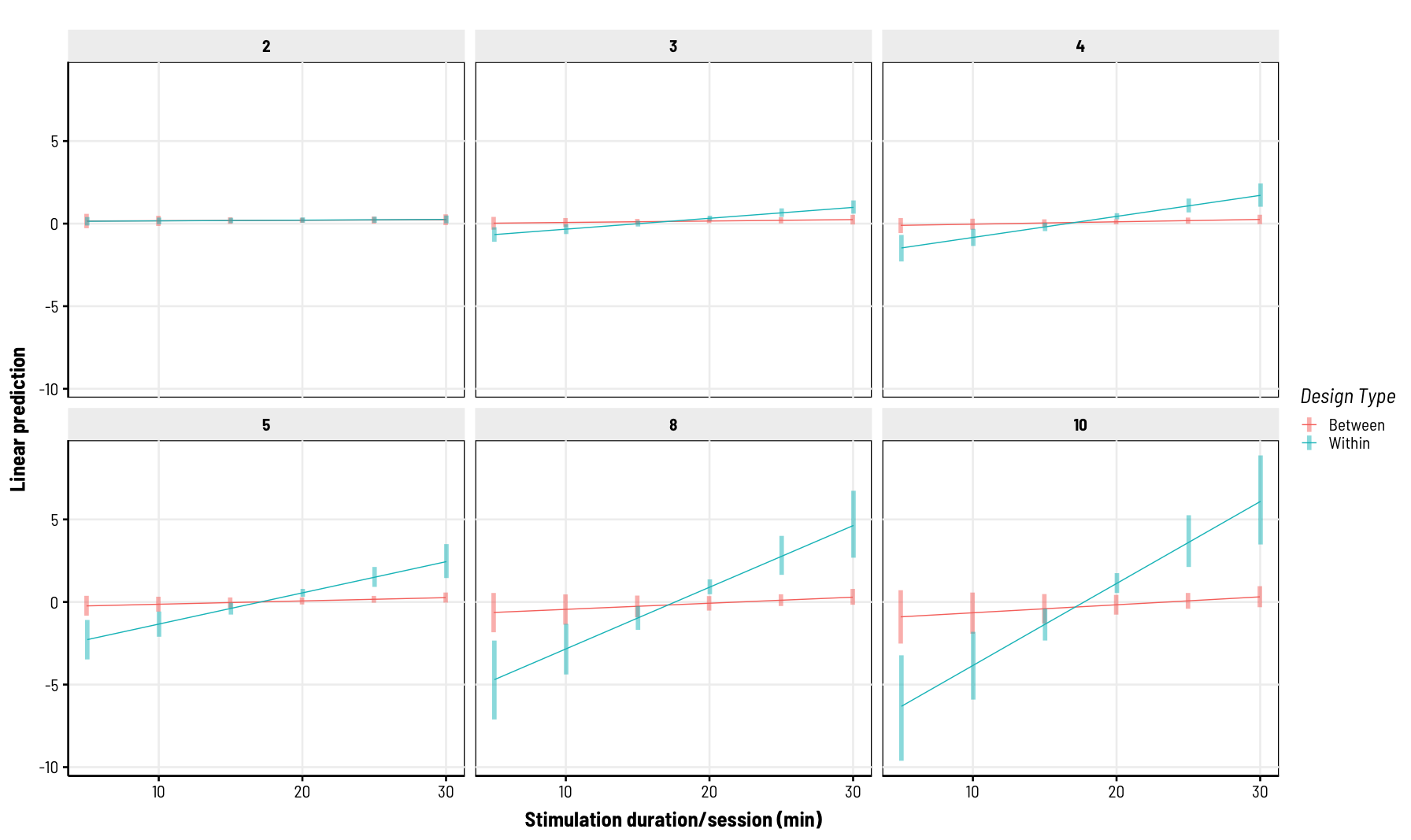


**Figure S4.** Posterior predictions for a three-way interaction between Design Type x Num Electrodes x Stim Duration (all domains combined). For visualization, stimulation durations (along the *x*-axis) were sampled at 5, 10, 15, 20, 25, and 30 minutes; montage sizes (individual facets/tiles) were sampled at 2, 3, 4, 5, 8, and 10 electrodes. Error bars give 95% highest posterior density intervals. These trends suggest that longer stimulation sessions with larger montages may benefit within-subjects designs while between-subjects designs are relatively invariant.

**Table S17.** Model summary for final candidate model with domains combined.

| **Fixed Predictors** | **Post. Med. Est.** | **Post. *SD*** | **95CI (*p*_d_)** |
| --- | --- | --- | --- |
| Intercept | 1.38 | 0.40 | 0.63 – 2.20 (1.00) |
| Polarity: Anodal - Cathodal | -0.08 | 0.04 | -0.16 – 0.00 (.977) |
| Laterality: Left - Bilateral | -0.20 | 0.10 | -0.39 – -0.01 (.980) |
| Laterality: Right - Bilateral | -0.18 | 0.09 | -0.36 – 0.01 (.970) |
| Number of stimulation sessions | 0.01 | 0.02 | -0.04 – 0.05 (.638) |
| Stimulation duration/session (min) | -0.06 | 0.02 | -0.10 – -0.03 (1.00) |
| Stimulation intensity (mA) | 0.01 | 0.07 | -0.13 – 0.16 (.554) |
| Stimulation timing: Offline - Mixed | 0.02 | 0.10 | -0.18 – 0.22 (.594) |
| Stimulation timing: Online - Mixed | -0.11 | 0.09 | -0.30 – 0.07 (.886) |
| Number of electrodes in montage | -0.64 | 0.16 | -0.97 – -0.34 (1.00) |
| Design: Within - Between | 1.89 | 0.75 | 0.48 – 3.44 (.996) |
| Design: Within - Between x  Number of electrodes in montage | -0.95 | 0.30 | -1.57 – -0.40 (1.00) |
| Design: Within - Between x  Stimulation duration/session | -0.11 | 0.04 | -0.19 – -0.04 (.999) |
| Number of electrodes in montage x  Stimulation duration/session | 0.03 | 0.01 | 0.02 – 0.05 (1.00) |
| Design: Within - Between x  Number of electrodes in montage x  Stimulation duration/session | 0.06 | 0.02 | 0.03 – 0.09 (1.00) |
| **Random Effects (*N* studies = 106)** | | | |
| σ_StudyID_ (Intercept) | 0.43 | 0.04 | 0.36 – 0.52 |
| *N* total effects = 721 | | | |
| Marginal *R*^2^ = 0.11 [0.06, 0.17] | | | |
| Conditional *R*^2^ = 0.32 [0.27, 0.36] | | | |
